# How to Escape Local Optima in Black Box Optimisation: When Non-elitism Outperforms Elitism

**DOI:** 10.1007/s00453-017-0369-2

**Published:** 2017-09-06

**Authors:** Pietro S. Oliveto, Tiago Paixão, Jorge Pérez Heredia, Dirk Sudholt, Barbora Trubenová

**Affiliations:** 1 University of Sheffield, Sheffield, S1 4DP UK; 2 IST Austria, Am Campus 1, 3400 Klosterneuburg, Austria

**Keywords:** Evolutionary algorithms, Runtime analysis, Population genetics, Strong selection weak mutation regime, Metropolis algorithm, Simulated annealing, Black box optimisation

## Abstract

Escaping local optima is one of the major obstacles to function optimisation. Using the metaphor of a fitness landscape, local optima correspond to hills separated by fitness valleys that have to be overcome. We define a class of fitness valleys of tunable difficulty by considering their *length*, representing the Hamming path between the two optima and their *depth*, the drop in fitness. For this function class we present a runtime comparison between stochastic search algorithms using different search strategies. The ($1+1$) EA is a simple and well-studied evolutionary algorithm that has to jump across the valley to a point of higher fitness because it does not accept worsening moves (elitism). In contrast, the Metropolis algorithm and the Strong Selection Weak Mutation (SSWM) algorithm, a famous process in population genetics, are both able to cross the fitness valley by accepting worsening moves. We show that the runtime of the ($1+1$) EA depends critically on the length of the valley while the runtimes of the non-elitist algorithms depend crucially on the depth of the valley. Moreover, we show that both SSWM and Metropolis can also efficiently optimise a rugged function consisting of consecutive valleys.

## Introduction

Black box algorithms are general purpose optimisation tools typically used when no good problem specific algorithm is known for the problem at hand. No particular knowledge is required for their application and they have been reported to be surprisingly effective. Popular classes are evolutionary algorithms, ant colony optimisation and artificial immune systems. These examples fall into the family of bio-inspired heuristics, but there are many other black box algorithms, including Simulated Annealing or Tabu Search. While many successful applications of these algorithms have been described, it is still hard to decide in advance which algorithm is preferable for a given problem. An initial natural research topic towards understanding the capabilities of a given algorithm is to identify classes of problems that are easy or hard for it [2, 6, 9, 13, 38]. However, the easiest and hardest classes of problems often are not closely related to real world applications. A more general question that applies to virtually any multimodal optimisation problem is to understand how efficient a given algorithm is in escaping from local optima.

Families of black box algorithms mainly differ in the way new solutions are generated (i.e. variation operators), how solutions are chosen for the next iterations (i.e. selection) and how many solutions are used by the heuristic in each iteration (i.e. population). Different variation operators, selection operators, population sizes and combinations of these lead to different algorithmic behaviours. In this paper we analyse the effects of mutation and selection in overcoming local optima.

Two different approaches are commonly used by most black box algorithms. One strategy is to rely on variation operators such as mutation to produce new solutions of high fitness outside the basin of attraction of the local optimum. These are unary operators that construct a new candidate solution typically by flipping bits of an existing solution. Elitist algorithms (i.e. those that never discard the best found solution), mainly rely on such strategies when stuck on a local optimum. In a population-based algorithm different individuals may use different mutation rates to help escape local optima faster [23]. Other variation operators may escape even faster than mutation. Population-based algorithms can recombine different solutions through the crossover operator to reach points outside the area of attraction of the local optima [14]. This operation requires that sufficient diversity is available in the population which may be introduced by using some diversity-enforcing mechanism [4]. Recently it has been shown that the interplay between the two variation operators, mutation and crossover, may efficiently give rise to the necessary burst of diversity without the need of any artificial diversity mechanism [3]. Another combination that has been proven to be effective for elitist algorithms to overcome local optima is to alternate mutations with variable depth search [35]. A common approach used in practice is to restart the algorithm or perform several runs in parallel with the hope that the algorithm does not get stuck on the same local optima every time.

A very different approach is to attempt to escape by accepting solutions of lower fitness in the hope of eventually leaving the basin of attraction of the local optimum. This approach is the main driving force behind non-elitist algorithms. Compared to the amount of work on elitist black box algorithms, there are few theoretical works analysing the performance of non-elitism (see, e. g. [5, 15, 16, 20, 21, 25, 26, 31, 33, 36]). While both approaches may clearly be promising, it is still unclear when one should be preferred to the other.

**Fig. 1:**
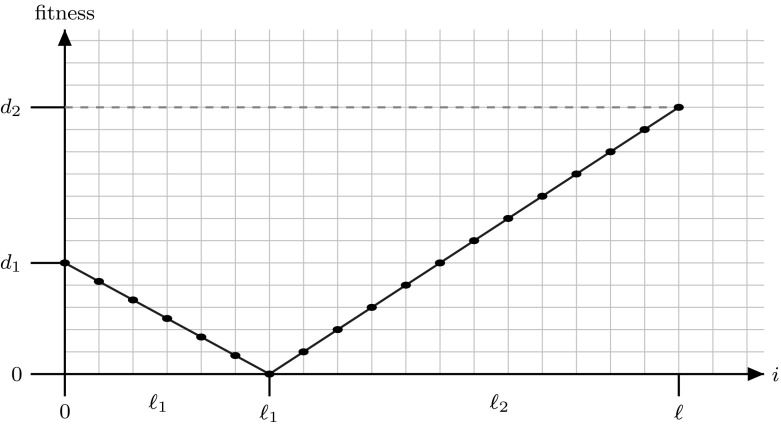
Sketch of the function $\textsc {Valley}$

In this paper we investigate this topic by considering the areas between consecutive local optima, which we call *fitness valleys*. These valleys can have arbitrary length $\ell$ i.e., the distance between the local optima, and arbitrary depth *d* i.e., the difference in function values between the optima and the point of minimum fitness between them. More precisely, we define a valley on a Hamming path (a path of Hamming neighbours) to ensure that mutation has the same probability of going forward on the path as going backwards. The valley is composed of a slope of length $\ell _1$ descending towards a local minimum from which a slope of increasing fitness of length $\ell _2$ can be taken to reach the end of the valley. The steepness of each slope is controlled by parameters $d_1$ and $d_2$, respectively indicating the fitness of the two local optima at the extreme left and extreme right of the valley. a sketch of a fitness valley is shown in Fig. 1. Our aim is to analyse how the characteristics of the valley impact the performance of elitist versus non-elitist strategies.

We point out that understanding how to cross fitness valleys efficiently is a very important problem also in biology [37]. From a biological perspective, crossing fitness valleys represents one of the major obstacles to the evolution of complex traits. Many of these traits require accumulation of multiple mutations that are individually harmful for their bearers; a fitness advantage is achieved only when all mutations have been acquired—a fitness valley has been crossed. We refer the interested reader to [27] for an attempt to unify evolutionary processes in computer science and population genetics.

We consider the simple elitist ($1+1$) EA, the most-studied elitist evolutionary algorithm, and compare its ability to cross fitness valleys with the recently introduced non-elitist Strong Selection Weak Mutation (SSWM) algorithm inspired by a model of biological evolution in the ‘strong selection, weak mutation regime’ [28, 29]. This regime applies when mutations are rare enough and selection is strong enough that the time between occurrences of new mutations is long compared to the time a new genotype takes to replace its parent genotype, or to be lost entirely [8]. Mutations occur rarely, therefore only one genotype is present in the population most of the time, and the relevant dynamics can be characterized by a stochastic process on one genotype. The significant difference between the SSWM algorithm and the ($1+1$) EA is that the former may accept solutions of lower quality than the current solution and even reject solutions of higher quality.

Recently, Paixão et al. investigated SSWM on $\text {Cliff}_{d}$ [28], a function defined such that non-elitist algorithms have a chance to jump down a “cliff” of height roughly *d* and to traverse a fitness valley of Hamming distance *d* to the optimum. The function is a generalised construction of the unitation function (a function that only depends on the number of 1-bits in the bit string) introduced by Jägersküpper and Storch to give an example class of functions where a (1, $\lambda$) EA outperforms a ($1+\lambda$) EA  [12]. This analysis revealed that SSWM can cross the fitness valley. However, upon comparison with the ($1+1$) EA, SSWM achieved only a small speed-up: the expected time (number of function evaluations) of SSWM is at most $n^{d}/e^{{\varOmega }(d)}$, while the ($1+1$) EA requires ${\varTheta }(n^d)$ [28].

In this manuscript, we show that greater speed-ups can be achieved by SSWM on fitness valleys. Differently to the work in [28] where global mutations were used, here we only allow SSWM to use local mutations because we are interested in comparing the benefits of escaping local optima by using non-elitism to cross valleys against the benefits of jumping to the other side by large mutations. Additionally, local mutations are a more natural variation operator for SSWM because they resemble more closely the biological processes from which the algorithm is inspired.

After presenting some Preliminaries, we build upon Gambler’s Ruin theory [7] in Sect. 3 to devise a general mathematical framework for the analysis of non-elitist algorithms using local mutations for crossing fitness valleys. We use it to rigorously show that SSWM is able to efficiently perform a random walk across the valley using only local mutations by accepting worse solutions, provided that the valley is not too deep. On the other hand, the ($1+1$) EA cannot accept worse solutions and therefore relies on global mutations to reach the other side of the valley in a single jump. More precisely, the ($1+1$) EA needs to make a jump across all valley points that have lower fitness; we call this the *effective length* of the valley.

As a result, the runtime of the ($1+1$) EA is exponential in the effective length of the valley while the runtime of SSWM depends crucially on the depth of the valley. We demonstrate the generality of the presented mathematical tool by using it to prove that the same asymptotic results achieved by SSWM also hold for the well-known Metropolis algorithm (simulated annealing with constant temperature) that, differently from SSWM, always accepts improving moves. Jansen and Wegener [15] previously compared the performance of the ($1+1$) EA and Metropolis for a fitness valley encoded as a unitation function where the slopes are symmetric and of the same length. They used their fitness valley as an example where the performance of the two algorithms is asymptotically equivalent.

The framework also allows the analysis for concatenated “paths” of several consecutive valleys, creating a rugged fitness landscape that loosely resembles a “big valley” structure found in many problems from combinatorial optimisation [1, 19, 22, 30]. In particular, in Sect. 4 we use it to prove that SSWM and Metropolis can cross consecutive paths in expected time that depends crucially on the depth and number of the valleys. Note that our preliminary work [24] required the more restrictive condition that the slope towards the optimum should be steeper than the one in the opposite direction i.e., $d_2/\ell _2 > d_1/\ell _1$. In this paper we have relaxed the conditions to consider only the depths of the valleys, i.e. $d_2 > d_1$. This generalisation allows the results to hold for a broader family of functions.

## Preliminaries

### Algorithms

In this paper we present a runtime comparison between the ($1+1$) EA and two non-elitist nature-inspired algorithms, SSWM and Metropolis. While they match the same basic scheme shown in Algorithm 1, they differ in the way they generate new solutions (mutate(*x*) function), and in the acceptance probability of these new solutions ($p_\mathrm {acc}$ function). 
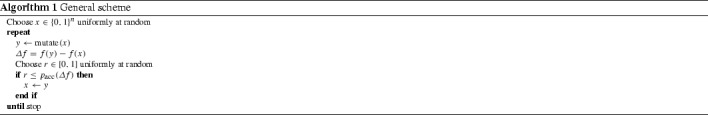
 The ($1+1$) EA relies on global mutations to cross the fitness valley and the function mutate(*x*) flips each bit independently with probability 1 / *n*. Conversely, SSWM and Metropolis analysed here use local mutations, hence the function mutate(*x*) flips a single bit chosen uniformly at random.

**Fig. 2:**
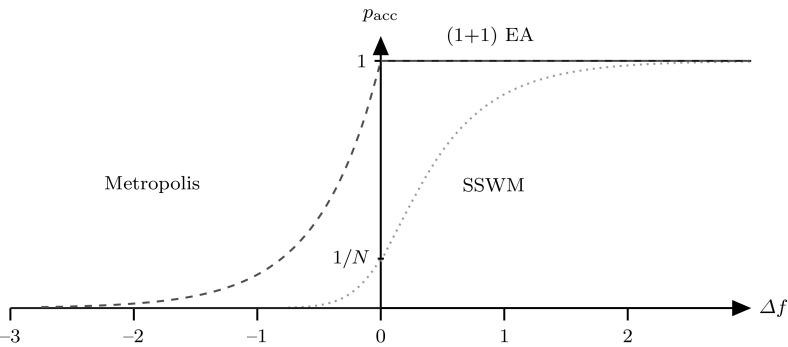
Probability of acceptance. *Red dashed line*—Metropolis, *Blue solid line*—($1+1$) EA, *Green dotted line*—SSWM (Color figure online)

Furthermore, the ($1+1$) EA always accepts a better solution, with ties resolved in favour of the new solution. The probability of acceptance is formally described by

$$\begin{aligned} p_\mathrm {acc}^{\mathrm {EA}}({\varDelta }f) = {\left\{ \begin{array}{ll} 1 &{} \text { if } {\varDelta }f \ge 0\\ 0 &{} \text { if } {\varDelta }f <0. \end{array}\right. } \end{aligned}$$

where ${\varDelta }f$ is the fitness difference between the new and the current solution. SSWM accepts candidate solutions with probability

1 $$\begin{aligned} p_\mathrm {acc}^{\mathrm {SSWM}}({\varDelta }f)=p_\mathrm {fix}({\varDelta }f)=\frac{1-e^{-2\beta {\varDelta }f}}{1-e^{-2 N\beta {\varDelta }f}} \end{aligned}$$

(see Fig. 2) where $N\ge 1$ is the size of the “population” that underlies the biological SSWM process as explained in the following paragraph, $\beta$ represents the *selection strength* and ${\varDelta }f \ne 0$. For ${\varDelta }f = 0$ we define $p_\mathrm {acc}(0) := \lim _{{\varDelta }f\rightarrow 0} p_\mathrm {acc}({\varDelta }f)=\frac{1}{N}$. If $N=1$, this probability is $p_\mathrm {acc}({\varDelta }f)=1$, meaning that any offspring will be accepted, and if $N\rightarrow \infty$, it will only accept solutions for which ${\varDelta }f>0$. SSWM’s acceptance function depends on the absolute difference in fitness between genotypes. It introduces two main differences compared to the ($1+1$) EA: first, solutions of lower fitness may be accepted with some positive probability, and second, solutions of higher fitness can be rejected.

Equation (1), first derived by Kimura [17], represents the probability that a gene that is initially present in one copy in a population of *N* individuals is eventually present in all individuals (the *probability of fixation*). Hence, Algorithm 1 takes a macro view to the adaptation process in that each iteration of the process models the appearance of a new mutation and its subsequent fate: either it is accepted with probability $p_\mathrm {acc}$, increasing to frequency 1 and replacing the previous genotype, or it is not and is lost. It is important to note that the *population size* *N* refers to the biological SSWM regime [29]. From the algorithmic perspective *N* is just a parameter of a single evolving individual.

The acceptance function $p_\mathrm {acc}$ is strictly increasing with the following limits: ${\lim _{{\varDelta }f\rightarrow -\infty }p_\mathrm {acc}({\varDelta }f)=0}$ and ${\lim _{{\varDelta }f\rightarrow \infty }p_\mathrm {acc}({\varDelta }f)=1}$. The same limits are obtained when $\beta$ tends to $\infty$, and thus for large $\vert \beta {\varDelta }f\vert$ the probability of acceptance is close to the one of the ($1+1$) EA, as long as $N>1$, defeating the purpose of the comparison, with the only difference being the tie-breaking rule: SSWM only accepts the new equally good solution with probability 1 / *N* [28].

Finally, the Metropolis algorithm is similar to SSWM in the sense that it is able to accept mutations that decrease fitness with some probability. However, unlike SSWM, for fitness improvements it behaves like the ($1+1$) EA in that it accepts *any* fitness improvement. Formally, Metropolis’ acceptance function can be described by:

2 $$\begin{aligned} p_\mathrm {acc}^{\mathrm {MET}}({\varDelta }f) = {\left\{ \begin{array}{ll} 1 &{} \text { if } {\varDelta }f \ge 0\\ e^{\alpha {\varDelta }f} &{} \text { if } {\varDelta }f <0 \end{array}\right. } \end{aligned}$$

where $\alpha$ is the reciprocal of the “temperature”. Temperature in the Metropolis algorithm plays the same role as population size in SSWM: increasing the temperature (decreasing $\alpha$) increases the probability of accepting fitness decreases. The acceptance functions of all three algorithms are shown in Fig. 2.

### Long Paths

Previous work on valley crossing [12, 15, 28] used functions of unitation to encode fitness valleys, with $1^n$ being a global optimum. The drawback of this construction is that the transition probabilities for mutation heavily depend on the current position. The closer an algorithm gets to $1^n$, the larger the probability of mutation decreasing the number of ones and moving away from the optimum.

We follow a different approach to avoid this mutational bias, and to ensure that the structure of the fitness valley is independent of its position in the search space. This also allows us to easily concatenate multiple valleys.

We base our construction on so-called *long k-paths*, paths of Hamming neighbours with increasing fitness whose length can be exponential in *n*. These paths were introduced and investigated experimentally in [11] and subsequently formalised and rigorously analysed in [32]. Exponential lower bounds were shown in [6]. An example of a long *k*-path is shown in Table 1. The following formal, slightly revised definition is taken from [34, p. 2517].

#### Definition 1

Let $k \in \mathbb {N}$ and *n* be a multiple of *k*. The long *k*-path of dimension *n* is a sequence of bit strings from $\{0, 1\}^n$ defined recursively as follows. The long *k*-path of dimension 0 is the empty bit string. Assume the long *k*-path of dimension $n-k$ is given by the sequence ${{\mathrm{\mathcal {P}}}}_{n-k}^k = (p_1, \dots , p_\ell )$, where $p_1, \dots , p_\ell \in \{0, 1\}^{n-k}$ and $\ell$ is the length of ${{\mathrm{\mathcal {P}}}}_{n-k}^k$. Then the long *k*-path of dimension *n* is defined by prepending *k* bits to these search points: let $S_0 := (0^k p_1, 0^k p_2, \dots , 0^k p_\ell )$, $S_1 := (1^k p_\ell , 1^k p_{\ell -1}, \dots , 1^k p_1)$, and $B := (0^{k-1}1 p_\ell , 0^{k-2}1^2 p_\ell , \dots , 01^{k-1} p_\ell )$. The search points in $S_0$ and $S_1$ differ in the *k* leading bits and the search points in *B* represent a bridge between them. The long *k*-path of dimension *n*, ${{\mathrm{\mathcal {P}}}}_n^k$, is the concatenation of $S_0, B$, and $S_1$.

The length of ${{\mathrm{\mathcal {P}}}}_n^k$ is $k \cdot 2^{n/k} - k + 1$ [34, Lemma 3], which is exponential in *n* if, for example, $k = {\varTheta }(\sqrt{n})$. An exponential length implies that the path has to be folded in $\{0, 1\}^n$ in a sense that there are $i < j$ such that the *i*-th and the *j*-th point on the path have Hamming distance $\text {H}(\cdot , \cdot )$ smaller than $j-i$. Standard bit mutations have a positive probability of jumping from the *i*-th to the *j*-th point, hence there is a chance to skip large parts of the path by taking a shortcut. However, long *k*-paths are constructed in such a way that at least *k* bits have to flip simultaneously in order to take a shortcut of length at least *k*. The probability of such an event is exponentially small if $k = {\varTheta }(\sqrt{n})$, in which case the path still has exponential length.

Long *k*-paths turn out to be very useful for our purposes. If we consider the first points of a long *k*-path and assign increasing fitness values to them, we obtain a fitness-increasing path of any desired length (up to exponential in *n* [34, Lemma 3]).

**Table 1:** Example of a long *k*-path for $n=9$ and $k=3$: ${{\mathrm{\mathcal {P}}}}_9^3 = (P_0, P_1, \dots , P_{21})$

$P_0:$	000000000	$P_6:$	000111111	$P_{12}:$	111111000	$P_{18}:$	111000111
$P_1:$	000000001	$P_7:$	000111011	$P_{13}:$	111111001	$P_{19}:$	111000011
$P_2:$	000000011	$P_8:$	000111001	$P_{14}:$	111111011	$P_{20}:$	111000001
$P_3:$	000000111	$P_9:$	000111000	$P_{15}:$	111111111	$P_{21}:$	111000000
$P_4:$	000001111	$P_{10}:$	001111000	$P_{16}:$	111011111		
$P_5:$	000011111	$P_{11}:$	011111000	$P_{17}:$	111001111		

Given two points ${{\mathrm{P}}}_s, {{\mathrm{P}}}_{s+i}$ for $i > 0$, ${{\mathrm{P}}}_{s+i}$ is called the *i*-th *successor* of ${{\mathrm{P}}}_s$ and ${{\mathrm{P}}}_s$ is called a *predecessor* of ${{\mathrm{P}}}_{s+i}$. Long *k*-paths have the following properties.

#### Lemma 1

(Long paths) 1. For every $i \in \mathbb {N}_0$ and path points ${{\mathrm{P}}}_s$ and ${{\mathrm{P}}}_{s+i}$, if $i < k$ then $\text {H}({{\mathrm{P}}}_s, {{\mathrm{P}}}_{s+i}) = i$, otherwise ${\text {H}({{\mathrm{P}}}_s, {{\mathrm{P}}}_{s+i}) \ge k}$.

2. The probability of a standard bit mutation turning ${{\mathrm{P}}}_s$ into ${{\mathrm{P}}}_{s+i}$ (or ${{\mathrm{P}}}_{s+i}$ into ${{\mathrm{P}}}_s$) is $1/n^i \cdot (1-1/n)^{n-i}$ for $0 \le i < k$ and the probability of reaching any search point in $\{{{\mathrm{P}}}_{s+i} \mid i \ge k\}$ from ${{\mathrm{P}}}_s$ is at most 1 / (*k*!).

#### Proof

The first statement was shown in [34, p. 2517] (refining a previous analysis in [6, p. 73]). The second statement follows from the first one, using that the probability of mutating at least *k* bits is at most $$\left( {\begin{array}{c}n\\ k\end{array}}\right) n^{-k} \le 1/(k!)$$. $\square$

In the following, we fix $k := \sqrt{n}$ such that the probability of taking a shortcut on the path is exponentially small. We assign fitness values such that all points on the path have a higher fitness than those off the path. This fitness difference is made large enough such that the considered algorithms are very unlikely to ever fall off the path. Assuming that we want to use the first *m* path points ${{\mathrm{P}}}_0, \dots , {{\mathrm{P}}}_{m-1}$, then the fitness is given by

$$\begin{aligned} f(x) := {\left\{ \begin{array}{ll} h(i) &{} \text {if }x = {{\mathrm{P}}}_i, i < m\\ -\infty &{} \text {otherwise} \end{array}\right. } \end{aligned}$$

where *h*(*i*) gives the fitness (height) of the *i*-th path point.

Then, assuming the algorithm is currently on the path, the fitness landscape is a one-dimensional landscape where (except for the two ends) each point has a Hamming neighbour as predecessor and a Hamming neighbour as successor on the path. Local mutations will create each of these with equal probability 1 / *n*. If we call these steps *relevant* and ignore all other steps, we get a stochastic process where in each relevant step we create a mutant up or down the path with probability 1 / 2 each (for the two ends we assume a self-loop probability of 1 / 2). The probability whether such a move is accepted then depends on the fitness difference between these path points.

It then suffices to study the expected number of relevant steps, as we obtain the expected number of function evaluations by multiplying with the expected waiting time *n* / 2 for a relevant step.

#### Lemma 2

Let $\text {E}\left( T\right)$ be the expected number of relevant steps for any algorithm described by Algorithm 1 with local mutations finding a global optimum. Then the respective expected number of function evaluations is $n/2 \cdot \text {E}\left( T\right)$, unless the algorithm falls off the path.

In the following, we assume that all algorithms start on ${{\mathrm{P}}}_0$. This behaviour can be simulated from random initialisation with high probability by embedding the path into a larger search space and giving hints to find the start of the path within this larger space [34]. As such a construction is cumbersome and does not lead to additional insights, we simply assume that all algorithms start in ${{\mathrm{P}}}_0$.

## Crossing Simple Valleys

On the first slope starting at point ${{\mathrm{P}}}_0$ the fitness decreases from the initial height $d_1 \in {\mathbb R}^+$ until the path point ${{\mathrm{P}}}_{\ell _1}$ with fitness 0. Then the second slope begins with fitness increasing up to the path point ${{\mathrm{P}}}_{\ell _1+\ell _2}$ of fitness $d_2 \in {\mathbb R}^+$. The total length of the path is $\ell =\ell _1+\ell _2$. We call such a path Valley.

$$\begin{aligned} h(i)_{\textsc {valley}} := {\left\{ \begin{array}{ll} d_1 - i \cdot \frac{d_1}{\ell _1} &{} \text { if }i \le \ell _1\\ (i-\ell _1) \cdot \frac{d_2}{\ell _2} &{} \text { if }\ell _1 < i \le \ell . \end{array}\right. } \end{aligned}$$

Here, $\frac{d_1}{\ell _1}$ and $\frac{d_2}{\ell _2}$ indicate the steepness of the two slopes (see Fig. 1). In this paper we will use $d_2>d_1$ to force the point ${{\mathrm{P}}}_{\ell }$ to be the optimum.

### Analysis for the ($1+1$) EA

We first show that the runtime of the ($1+1$) EA depends on the effective length $\ell ^*$ of the valley, defined as the distance between the initial point ${{\mathrm{P}}}_0$ and the first valley point of greater or equal fitness. Here we restrict parameters to $\ell _1 + \ell _2 \le \sqrt{n}/4$, as then the probability of the ($1+1$) EA taking a shortcut is no larger than the probability of jumping by a distance of $\ell _1+\ell _2$: $\frac{1}{(\sqrt{n})!} \le n^{-\sqrt{n}/4}$ for $n \ge 4$.

#### Theorem 3

Assume $\ell _1 + \ell _2 \le \sqrt{n}/4$ and $d_1 \le d_2$. The expected time for the ($1+1$) EA starting in ${{\mathrm{P}}}_0$ to cross the fitness valley is ${\varTheta }(n^{\ell ^*})$ where $\ell ^* = \ell _1+\lceil d_1\ell _2/d_2\rceil$.

#### Proof

Let us first recall that due to its elitism the ($1+1$) EA can not fall off the path. To cross the fitness valley the ($1+1$) EA needs to jump from $P_0$ to a point with higher fitness, thus it has to jump at least a distance $\ell ^*$. The probability of such a jump can be bounded from below using Lemma 1 by

3 $$\begin{aligned} p_{\mathrm {jump}}&\ge n^{-\ell ^*} \left( 1-\frac{1}{n}\right) ^{n-\ell ^*} \ge e^{-1}n^{-\ell ^*} \end{aligned}$$

resulting in an expected time needed to jump over the valley of at most $en^{\ell ^*} = O(n^{\ell ^*})$. After jumping over the valley, the ($1+1$) EA has to climb at most the remaining $\left( 1-\frac{d_1}{d_2}\right) \ell _2 \le \ell _2$ steps, and each improvement has a probability of at least 1 / (*en*). The expected time for this climb is thus at most $e\ell _2n$. As $\ell _2<n$ and ${\ell ^*} \ge \ell _1 \ge 2$, this time is $O(n^{\ell ^*})$.

Note that, in case ${{\mathrm{P}}}_{\ell ^*}$ has the same fitness as ${{\mathrm{P}}}_0$, the ($1+1$) EA can jump back to the beginning of the path, in which case it needs to repeat the jump. However, conditional on leaving ${{\mathrm{P}}}_{\ell ^*}$, the probability that a successor is found is at least ${\varOmega }(1)$. Hence in expectation *O*(1) jumps are sufficient.

Furthermore, the probability of the jump can be bounded from above by the probability of jumping to any of the next potential $\sqrt{n}$ path points and by the probability of taking a shortcut (see Lemma 1)

$$\begin{aligned} p_{\mathrm {jump}}&\le \frac{1}{\sqrt{n}!} + \sum _{i={\ell ^*}}^{\sqrt{n}} n^{-i}\left( 1-\frac{1}{n}\right) ^{n-i} = O\left( n^{-{\ell ^*}}\right) \end{aligned}$$

where we used $\frac{1}{(\sqrt{n})!} \le n^{-\sqrt{n}/4} \le n^{-{\ell ^*}}$. Thus the expected time is ${\varOmega }(n^{\ell ^*})$. $\square$

### A General Framework for Local Search Algorithms

We introduce a general framework to analyse the expected number of relevant steps of non-elitist local search algorithms (Algorithm 1 with local mutations) for the Valley problem. As explained in Sect. 2.2, in a relevant step mutation creates a mutant up or down the path with probability 1 / 2, and this move is accepted with a probability that depends only on the fitness difference. For slopes where the gradient is the same at every position, this resembles a gambler’s ruin process.

To apply classical gambler ruin theory (see e.g. [7]) two technicalities need to be taken into account. Firstly, two different gambler ruin games need to be considered, one for descending down the first slope and another one for climbing up the second slope. The process may alternate between these two ruin games as the extreme ends of each game at the bottom of the valley are not absorbing states. Secondly, a non-elitist algorithm could reject the offspring individual even when it has a higher fitness than its parent. Hence the probabilities of winning or losing a dollar (i.e., the probabilities of moving one step up or down in the slope) do not necessarily add up to one, but loop probabilities of neither winning or losing a dollar need to be taken into account when estimating expected times (winning probabilities are unaffected by self-loops).

#### Theorem 4

(Gambler’s Ruin with self-loops) Consider a game where two players start with $n_1 \in {\mathbb N}^+$ and $n_2 \in {\mathbb N}^+$ dollars respectively. In each iteration player 1 wins one of player’s 2 dollars with probability $p_1$, player 2 wins one of player’s 1 dollars with probability $p_2$, and nothing happens with probability $1-p_1-p_2$. Then the probability of player 1 winning all the dollars before going bankrupt is:

$$\begin{aligned} P_1 = {\left\{ \begin{array}{ll} \frac{n_1}{n_1+n_2} &{} \text { if }p_1 = p_2 \\ \frac{1-\left( \frac{p_2}{p_1}\right) ^{n_1}}{1-\left( \frac{p_2}{p_1}\right) ^{n_1+n_2}} \;\; &{} \text { if }p_1 \ne p_2. \end{array}\right. } \end{aligned}$$

The expected time until either of both players become bankrupt i.e. the expected duration of the game is

$$\begin{aligned} \text {E}\left( T\right) = {\left\{ \begin{array}{ll} \frac{n_1 n_2}{p_1+p_2} &{} \text { if }p_1 = p_2\\ \frac{n_1-(n_1+n_2)P_1}{p_2^2-p_1^2} \;\; &{} \text { if }p_1 \ne p_2. \end{array}\right. } \end{aligned}$$

#### Proof

The proof follows directly from the results of the standard problem ($p_1+p_2=1$) see e.g. Chapter XIV in [7]. The only effect of the self-loops is to add extra iterations in the problem where nothing happens, therefore the winning probabilities will not be affected, however the expected duration of the game will be increased by the waiting time needed for a relevant iteration $1/(p_1+p_2)$. $\square$

In order to simplify the calculations we have developed the following notation.

#### Definition 2

(*Framework’s notation*) The Valley problem can be considered as a Markov chain with states $\{P_0,P_1,\dots ,P_{\ell _1-1},P_{\ell _1},P_{\ell _1+1},\dots ,P_{\ell _1+\ell _2}\}.$ For simplicity we will sometimes refer to these points only with their sub-indices $\{0,1,\dots ,\ell _1-1,\ell _1,\ell _1+1,\dots ,\ell _1+\ell _2\}$. For any stochastic process on the Valley problem we will denote by:

1. $p_{i \rightarrow j}$ the probability of moving from state *i* to $j\in \{i-1,i,i+1\}$ in one iteration,
2. $p^\mathrm {GR}_{i \rightarrow k}$ the probability of a Gambler’s Ruin process starting in *i* finishing in *k* before reaching the state $i-1$,
3. $\text {E}\left( T^\mathrm {GR}_{i , k}\right)$ the expected duration until either the state $i-1$ or *k* is reached,
4. $\text {E}\left( T_{i\rightarrow m}\right)$ the expected time to move from state *i* to state *m*.

The following lemmas simplify the runtime analysis of any algorithm that matches the scheme of Algorithm 1 for local mutations and some reasonable conditions on the selection operator.

#### Lemma 5

Consider any algorithm described by Algorithm 1 with local mutations and the following properties on Valley with $\ell _1, \ell _2 \in \{2,3,\dots \}$ and $d_1, d_2 \in R^+$

(i) $$p_{\ell _1 \rightarrow \ell _1-1},\;p_{\ell _1 \rightarrow \ell _1+1} = {\varOmega }(1)$$
(ii) $p_{\ell _1 \rightarrow \ell _1+1} \ge \; p_{\ell _1+1 \rightarrow \ell _1}+\varepsilon$, for $\varepsilon >0$ a constant
(iii) $p_{\mathrm {acc}}({\varDelta }f)$ is non-decreasing.

Then the expected number of relevant steps for such a process to reach the point $P_{\ell _1+\ell _2}$ starting from $P_{0}$ is

$$\begin{aligned} \text {E}\left( T_{0\rightarrow \ell _1+\ell _2}\right) = {\varTheta }\left( \text {E}\left( T_{1\rightarrow \ell _1}\right) \right) + {\varTheta }(\ell _2). \end{aligned}$$

Property (iii) describes a common feature of optimisation algorithms: the selection operator prefers fitness increases over decreases (e.g. Randomised Local Search, ($1+1$) EA or Metropolis). Then, the bottleneck of Valley seems to be climbing down the first $\ell _1$ steps since several fitness decreasing mutations have to be accepted.

Once at the bottom of the valley $P_{\ell _1}$ the process must keep moving. It could be the case that the algorithm climbs up again to $P_0$. But under some mild conditions it will only have to repeat the experiment a constant number of times (property (i) of the following lemma).

Finally, the algorithm will have to climb up to $P_{\ell _1+\ell _2}$. This will take linear time in $\ell _2$, provided the probability of accepting an improvement $p_{\ell _1 \rightarrow \ell _1+1}$ is by a constant greater than accepting a worsening of the same size $p_{\ell _1+1 \rightarrow \ell _1}$, as required by property (ii).

Consider an algorithm with a selection operator that satisfies condition (iii) such as Metropolis or SSWM. In order to satisfy the first two conditions, the selection strength must be big enough to accept the two possible fitness increases of Valley ($d_1/\ell _1$ and $d_2/\ell _2$) with constant probability. As we will see at the end of this section, this condition directly translates to $\beta d_1/\ell _1,\;\beta d_2/\ell _2={\varOmega }(1)$ for SSWM and $\alpha d_1/\ell _1,\;\alpha d_2/\ell _2={\varOmega }(1)$ for Metropolis.

In order to prove the previous lemma we will make use of the following lemma that shows some implications of the conditions from the previous lemma.

#### Lemma 6

In the context of Lemma 5, properties (i) and (ii) imply that

(i) $p_{\ell _1 \rightarrow \ell _1-1}+p_{\ell _1 \rightarrow \ell _1+1} = 1/c_1$ for some constant $c_1\ge 1$
(ii) $1-c_1\cdot p^\mathrm {GR}_{\ell _1+1 \rightarrow \ell _1} = 1/c_2$ for some constant $c_2>1$
(iii) $1-c_1c_2\cdot p_{\ell _1 \rightarrow \ell _1-1} = 1/c_3$ for some constant $c_3>1$.

For the sake of readability the proof of Lemma 6 can be found in the appendix.

#### Proof of Lemma 5

Since the algorithm only produces points in the Hamming neighbourhood it will have to pass through all the states on the path. We break down the set of states in three sets and expand the total time as the sum of the optimisation time for those three sets:

4 $$\begin{aligned} \text {E}\left( T_{0 \rightarrow \ell _1+\ell _2}\right) = \text {E}\left( T_{0 \rightarrow 1}\right) + \text {E}\left( T_{1 \rightarrow \ell _1}\right) + \text {E}\left( T_{\ell _1 \rightarrow \ell _1+\ell _2}\right) . \end{aligned}$$

Note that the lower bound follows directly. Let us now consider the upper bound. We start using a recurrence relation for the last term: once in state $\ell _1$, after one iteration, the algorithm can either move to state $\ell _1+1$ with probability $p_{\ell _1 \rightarrow \ell _1+1}$, move to state $\ell _1-1$ with probability $p_{\ell _1 \rightarrow \ell _1-1}$ or stay in state $\ell _1$ with the remaining probability (if the mutation is not accepted).

$$\begin{aligned} \text {E}\left( T_{\ell _1 \rightarrow \ell _1+\ell _2}\right)&= 1 + p_{\ell _1 \rightarrow \ell _1+1} \cdot \text {E}\left( T_{\ell _1+1 \rightarrow \ell _1+\ell _2}\right) \\&\quad \quad + p_{\ell _1 \rightarrow \ell _1-1} \cdot \text {E}\left( T_{\ell _1-1 \rightarrow \ell _1+\ell _2}\right) + p_{\ell _1 \rightarrow \ell _1}\cdot \text {E}\left( T_{\ell _1 \rightarrow \ell _1+\ell _2}\right) . \end{aligned}$$

Using $\text {E}\left( T_{\ell _1-1 \rightarrow \ell _1+\ell _2}\right) \le \text {E}\left( T_{0 \rightarrow \ell _1+\ell _2}\right)$ this expression reduces to

$$\begin{aligned}&\le 1 + p_{\ell _1 \rightarrow \ell _1+1} \cdot \text {E}\left( T_{\ell _1+1 \rightarrow \ell _1+\ell _2}\right) +\quad p_{\ell _1 \rightarrow \ell _1-1} \cdot \text {E}\left( T_{0 \rightarrow \ell _1+\ell _2}\right) \\&\quad + p_{\ell _1 \rightarrow \ell _1}\cdot \text {E}\left( T_{\ell _1 \rightarrow \ell _1+\ell _2}\right) . \end{aligned}$$

Solving the previous expression for $\text {E}\left( T_{\ell _1 \rightarrow \ell _1+\ell _2}\right)$ leads to

$$\begin{aligned}&\text {E}\left( T_{\ell _1 \rightarrow \ell _1+\ell _2}\right) \le \frac{1 + p_{\ell _1 \rightarrow \ell _1+1} \cdot \text {E}\left( T_{\ell _1+1 \rightarrow \ell _1+\ell _2}\right) + p_{\ell _1 \rightarrow \ell _1-1} \cdot \text {E}\left( T_{0 \rightarrow \ell _1+\ell _2}\right) }{p_{\ell _1 \rightarrow \ell _1-1}+p_{\ell _1 \rightarrow \ell _1+1}}. \end{aligned}$$

Since property (i) of Lemma 5 implies that the denominator is a constant $1/c_1$, we get

5 $$\begin{aligned}&\text {E}\left( T_{\ell _1 \rightarrow \ell _1+\ell _2}\right) \le c_1 \left( 1 + p_{\ell _1 \rightarrow \ell _1+1} \cdot \text {E}\left( T_{\ell _1+1 \rightarrow \ell _1+\ell _2}\right) + p_{\ell _1 \rightarrow \ell _1-1} \cdot \text {E}\left( T_{0 \rightarrow \ell _1+\ell _2}\right) \right) . \end{aligned}$$

Let us now focus on the term $\text {E}\left( T_{\ell _1+1 \rightarrow \ell _1+\ell _2}\right)$. Since the acceptance probability is a function of ${\varDelta }f$, for both sides of the valley the probabilities of moving to the next or previous state remain constant during each slope and we can cast the behaviour as a Gambler’s Ruin problem. Then, when the state is $P_{\ell _1+1}$ a Gambler’s Ruin game (with self-loops) occurs. The two possible outcomes are: (1) the problem is optimised or (2) we are back in $P_{\ell _1}$. Hence,

6 $$\begin{aligned} \text {E}\left( T_{\ell _1+1 \rightarrow \ell _1+\ell _2}\right) = \text {E}\left( T^\mathrm {GR}_{\ell _1+1, \ell _1+\ell _2}\right) + p^\mathrm {GR}_{\ell _1+1 \rightarrow \ell _1} \cdot \text {E}\left( T_{\ell _1 \rightarrow \ell _1+\ell _2}\right) . \end{aligned}$$

Now we introduce (6) in (5), obtaining

$$\begin{aligned} \text {E}\left( T_{\ell _1 \rightarrow \ell _1+\ell _2}\right)&\le c_1\left( 1 + p_{\ell _1 \rightarrow \ell _1-1} \cdot \text {E}\left( T_{0 \rightarrow \ell _1+\ell _2}\right) \right) \\&\quad + c_1 \cdot p_{\ell _1 \rightarrow \ell _1+1} \cdot \left( \text {E}\left( T^\mathrm {GR}_{\ell _1+1 , \ell _1+\ell _2}\right) + p^\mathrm {GR}_{\ell _1+1 \rightarrow \ell _1} \cdot \text {E}\left( T_{\ell _1 \rightarrow \ell _1+\ell _2}\right) \right) . \end{aligned}$$

Solving for $\text {E}\left( T_{\ell _1 \rightarrow \ell _1+\ell _2}\right)$ yields

$$\begin{aligned}&\text {E}\left( T_{\ell _1 \rightarrow \ell _1+\ell _2}\right) \le \frac{c_1 \left( 1 + p_{\ell _1 \rightarrow \ell _1+1} \cdot \text {E}\left( T^\mathrm {GR}_{\ell _1+1 , \ell _1+\ell _2}\right) + p_{\ell _1 \rightarrow \ell _1-1} \cdot \text {E}\left( T_{0 \rightarrow \ell _1+\ell _2}\right) \right) }{1-c_1\cdot p_{\ell _1 \rightarrow \ell _1+1} \cdot p^\mathrm {GR}_{\ell _1+1 \rightarrow \ell _1} }. \end{aligned}$$

By Lemma 6, properties (i) and (ii) of Lemma 5 imply that the denominator is a constant $1/c_2$. Hence,

$$\begin{aligned} \text {E}\left( T_{\ell _1 \rightarrow \ell _1+\ell _2}\right)&\le c_1c_2\cdot \left( 1 + p_{\ell _1 \rightarrow \ell _1+1} \cdot \text {E}\left( T^\mathrm {GR}_{\ell _1+1 , \ell _1+\ell _2}\right) + p_{\ell _1 \rightarrow \ell _1-1} \cdot \text {E}\left( T_{0 \rightarrow \ell _1+\ell _2}\right) \right) \\&\le c_1c_2\cdot \left( 1 + \text {E}\left( T^\mathrm {GR}_{\ell _1+1 , \ell _1+\ell _2}\right) + p_{\ell _1 \rightarrow \ell _1-1} \cdot \text {E}\left( T_{0 \rightarrow \ell _1+\ell _2}\right) \right) . \end{aligned}$$

We introduce this into (4), leading to

$$\begin{aligned} \text {E}\left( T_{0 \rightarrow \ell _1+\ell _2}\right)&\le \; \text {E}\left( T_{0 \rightarrow 1}\right) + \text {E}\left( T_{1 \rightarrow \ell _1}\right) \\&\qquad +c_1c_2 \left( 1 + \text {E}\left( T^\mathrm {GR}_{\ell _1+1 , \ell _1+\ell _2}\right) + p_{\ell _1 \rightarrow \ell _1-1} \cdot \text {E}\left( T_{0 \rightarrow \ell _1+\ell _2}\right) \right) . \end{aligned}$$

Solving for $\text {E}\left( T_{0 \rightarrow \ell _1+\ell _2}\right)$ leads to

$$\begin{aligned} \text {E}\left( T_{0 \rightarrow \ell _1+\ell _2}\right)&\le \; \frac{\text {E}\left( T_{0 \rightarrow 1}\right) + \text {E}\left( T_{1 \rightarrow \ell _1}\right) + c_1c_2 + c_1c_2\cdot \text {E}\left( T^\mathrm {GR}_{\ell _1+1 , \ell _1+\ell _2}\right) }{1-c_1c_2\cdot p_{\ell _1 \rightarrow \ell _1-1}}. \end{aligned}$$

Again by Lemma 6, properties (i) and (ii) of Lemma 5 imply that the denominator is a constant $1/c_3$. Hence,

7 $$\begin{aligned} \text {E}\left( T_{0 \rightarrow \ell _1+\ell _2}\right) \le c_3 \left( \text {E}\left( T_{0 \rightarrow 1}\right) + \text {E}\left( T_{1 \rightarrow \ell _1}\right) + c_1c_2 + c_1c_2\cdot \text {E}\left( T^\mathrm {GR}_{\ell _1+1 , \ell _1+\ell _2}\right) \right) . \end{aligned}$$

Since $\text {E}\left( T_{0 \rightarrow 1}\right) \le \text {E}\left( T_{1 \rightarrow \ell _1}\right)$ we have that $\text {E}\left( T_{0 \rightarrow 1}\right) + \text {E}\left( T_{1 \rightarrow \ell _1}\right) = {\varTheta }\left( \text {E}\left( T_{1 \rightarrow \ell _1}\right) \right)$. Now we consider the last term. Due to property (ii) of Lemma 5, once in $\ell _1+1$ there is a constant probability of moving towards the optimum. Since the algorithm has to cover a distance of $\ell _2+\ell _1 - (\ell _1+1) = \ell _2 -1$, then $\text {E}\left( T^\mathrm {GR}_{\ell _1+1 , \ell _1+\ell _2}\right) = {\varTheta }(\ell _2)$. Plugging this into (7) proves the claimed upper bound. $\square$

Now we estimate the time to move from $P_0$ to $P_{\ell _1}$. As in the previous proof, the main arguments are a recurrence relation and a Gambler’s Ruin game.

#### Lemma 7

Consider any algorithm described by Algorithm 1 with local mutations on Valley with $\ell _1, \ell _2 \in {\mathbb N}{\setminus }\{1\}$ and $d_1, d_2 \in R^+$. Then the number of relevant steps to go from the state $P_1$ to $P_{\ell _1}$ is

$$\begin{aligned} \text {E}\left( T_{1 \rightarrow \ell _1}\right) = \frac{1}{p^\mathrm {GR}_{1 \rightarrow \ell _1}} \cdot \left( \text {E}\left( T^\mathrm {GR}_{1 , \ell _1}\right) + \frac{p^\mathrm {GR}_{1 \rightarrow 0}}{p_{0 \rightarrow 1}} \right) . \end{aligned}$$

#### Proof

At the state $P_1$ a Gambler’s Ruin game (with self-loops) occurs. The two possible outcomes are: (1) we have reached the valley $P_{\ell _1}$ or (2) we are back to $P_{0}$. Hence,

$$\begin{aligned} \text {E}\left( T_{1 \rightarrow \ell _1}\right) =\;&\text {E}\left( T^\mathrm {GR}_{1 , \ell _1}\right) + p^\mathrm {GR}_{1 \rightarrow 0}\cdot \text {E}\left( T_{0 \rightarrow \ell _1}\right) \\ =\;&\text {E}\left( T^\mathrm {GR}_{1 , \ell _1}\right) + p^\mathrm {GR}_{1 \rightarrow 0}\cdot \left( \text {E}\left( T_{0 \rightarrow 1}\right) + \text {E}\left( T_{1 \rightarrow \ell _1}\right) \right) . \end{aligned}$$

Solving for $\text {E}\left( T_{1 \rightarrow \ell _1}\right)$ leads to

$$\begin{aligned} \text {E}\left( T_{1 \rightarrow \ell _1}\right) =\;&\frac{\text {E}\left( T^\mathrm {GR}_{1 , \ell _1}\right) + p^\mathrm {GR}_{1 \rightarrow 0}\cdot \text {E}\left( T_{0 \rightarrow 1}\right) }{1-p^\mathrm {GR}_{1 \rightarrow 0}}, \end{aligned}$$

which, by using $1-p^\mathrm {GR}_{1 \rightarrow 0}=p^\mathrm {GR}_{1 \rightarrow \ell _1}$, simplifies to

8 $$\begin{aligned} \text {E}\left( T_{1 \rightarrow \ell _1}\right) =\;&\frac{1}{p^\mathrm {GR}_{1 \rightarrow \ell _1}} \cdot \left( \text {E}\left( T^\mathrm {GR}_{1 , \ell _1}\right) + \frac{p^\mathrm {GR}_{1 \rightarrow 0}}{p_{0 \rightarrow 1}} \right) . \end{aligned}$$

$$\square $$

### Application to SSWM

In this subsection we make use of the previous framework to analyse the SSWM for the Valley problem. To apply this framework we need to know how a Gambler’s Ruin with the acceptance probabilities of the SSWM behaves. When dealing with these probabilities the ratio between symmetric fitness variations appears often. The next lemma will be very helpful to simplify this ratio.

#### Lemma 8

(Lemma 2 in [28]) For every $\beta \in {\mathbb R}^+$, ${\varDelta }f \in {\mathbb R}$ and $N \in {\mathbb N}^+$

$$\begin{aligned} \frac{p_\mathrm {fix}(-{\varDelta }f)}{p_\mathrm {fix}(+{\varDelta }f)} = e^{-2(N-1)\beta {\varDelta }f}. \end{aligned}$$

#### Proof

The proof follows from the definition of $p_\mathrm {fix}$ [see Eq. (1)] and applying the relation $e^x=(e^x-1)/(1-e^{-x})$. $\square$

Due to the sigmoid expression of the SSWM acceptance probability [Eq. (1)], it can be helpful to use bounds given by simpler expressions. Lemma 1 in [28] provides such bounds.

#### Lemma 9

(Lemma 1 in [28]) For every $\beta \in {\mathbb R}^+$ and $N \in {\mathbb N}^+$ the following inequalities hold. If ${\varDelta }f \ge 0$ then

$$\begin{aligned} \frac{2\beta {\varDelta }f}{1+2\beta {\varDelta }f} \le p_\mathrm {fix}({\varDelta }f) \le \frac{2\beta {\varDelta }f}{1-e^{-2N\beta {\varDelta }f}}. \end{aligned}$$

If $${\varDelta }f \le 0$$ then

$$\begin{aligned} \frac{-2\beta {\varDelta }f }{e^{-2 N \beta {\varDelta }f}}\le p_\mathrm {fix}({\varDelta }f) \le \frac{e^{-2\beta {\varDelta }f}}{e^{-2N\beta {\varDelta }f}-1}. \end{aligned}$$

The following lemma contains bounds on the expected duration of the game and winning probabilities for SSWM. Although Valley has slopes of $d_1/\ell _1$ and $d_2/\ell _2$, SSWM through the action of the parameter $\beta$ sees an effective gradient of $\beta \cdot d_1/\ell _1$ and $\beta \cdot d_2/\ell _2$. Varying this parameter allows the algorithm to accommodate the slope to a comfortable value. We have set this effective gradient to $\beta |{\varDelta }f| = {\varOmega }(1)$ so that the probability of accepting an improvement is at least a constant.

#### Lemma 10

(SSWM Gambler’s Ruin) Consider a Gambler’s Ruin problem as described in Theorem 4 with starting dollars $n_1=1$ and $n_2=\ell -1$, and probabilities $p_1$ and $p_2$ dependant on SSWM’s acceptance function as follows

$$\begin{aligned} p_1 = \frac{1}{2}\cdot p_\mathrm {fix}({\varDelta }f) \;\;\;\;\;\ p_2 = \frac{1}{2}\cdot p_\mathrm {fix}(-{\varDelta }f) \end{aligned}$$

where ${\varDelta }f < 0$ and $(N-1)\beta |{\varDelta }f| = {\varOmega }(1)$. Then the winning probability of player one $P^{\mathrm {GR}}_{1\rightarrow \ell _1}$ can be bounded as follows

$$\begin{aligned} \frac{-2(N-1)\beta {\varDelta }f}{e^{-2(N-1)\beta (n_1+n_2) {\varDelta }f}} \le \; P^{\mathrm {GR}}_{1\rightarrow \ell _1} \;\le \frac{e^{-2(N-1)\beta {\varDelta }f}}{e^{-2(N-1)\beta (n_1+n_2) {\varDelta }f}-1} \end{aligned}$$

and the expected duration of the game will be $\text {E}\left( T^\mathrm {GR}_{1 , \ell }\right) = O(1)$.

#### Proof

We start with the winning probability. Invoking Theorem 4 and simplifying the ratio of $p_\mathrm {fix}$ of symmetric fitness variations with Lemma 8 we obtain

$$\begin{aligned} P^{\mathrm {GR}}_{1\rightarrow \ell _1} = \frac{1-\left( \frac{p_2}{p_1}\right) ^{n_1}}{1-\left( \frac{p_2}{p_1} \right) ^{n_1+n_2}} = \frac{1-\left( \frac{p_\mathrm {fix}(-{\varDelta }f)}{p_\mathrm {fix}({\varDelta }f)}\right) ^{n_1}}{1-\left( \frac{p_\mathrm {fix}(-{\varDelta }f)}{p_\mathrm {fix}({\varDelta }f)} \right) ^{n_1+n_2}} = \frac{1-e^{-2(N-1)\beta n_1 {\varDelta }f}}{1-e^{-2(N-1)\beta (n_1+n_2) {\varDelta }f}}. \end{aligned}$$

Notice that this is the same expression as the acceptance probability if we change $\beta$ for $(N-1)\beta$ and *N* for $\ell$. Then we can apply the bounds for the original acceptance probabilities from Lemma 9 to obtain the inequalities of the theorem’s statement.

Finally, for the expected duration of the game we call again Theorem 4

$$\begin{aligned} \text {E}\left( T^\mathrm {GR}_{1 , \ell }\right)&= \frac{1}{p_2+p_1}\cdot \frac{ n_1 - (n_1+n_2)\cdot P^{\mathrm {GR}}_{1\rightarrow \ell _1}}{p_2-p_1} \\&\le \; \frac{ 1 - \ell \cdot P^{\mathrm {GR}}_{1\rightarrow \ell _1}}{p_2^2-p_1^2} \le \; \frac{1}{p_2^2-p_1^2} = \frac{1}{p_2^2 \left( 1- \frac{p_1^2}{p_2^2} \right) }\\&= \frac{1}{p_2^2 \left( 1- e^{-4\beta (N-1){\varDelta }f} \right) }. \end{aligned}$$

Note that in the last step we have used Lemma 8, and that since $N\ge 2$ the condition $(N-1)\beta |{\varDelta }f|={\varOmega }(1)$ implies that $\beta |{\varDelta }f|={\varOmega }(1)$. Hence all the parameters of SSWM’s acceptance probability [Eq. (1)] are ${\varOmega }(1)$ and so is $p_2$. For the same reason the factor $1- e^{-4\beta (N-1){\varDelta }f}$ is constant yielding $\text {E}\left( T^\mathrm {GR}_{1 , \ell }\right) =O(1)$. $\square$

While the optimisation time of the ($1+1$) EA grows exponentially with the length of the valley, the following theorem shows that for the SSWM the growth is exponential in the depth of the valley.

#### Theorem 11

The expected number of function evaluations $\text {E}\left( T_f\right)$ for SSWM with local mutations to reach ${{\mathrm{P}}}_{\ell _1+\ell _2}$ from ${{\mathrm{P}}}_0$ on Valley with $\ell _1, \ell _2 \in \{2,3,\dots \}$ and $d_1, d_2 \in R^+$ is

$$\begin{aligned} \text {E}\left( T_f\right)&= O\left( n \cdot e^{2N\beta d_1 (\ell _1+1)/\ell _1} \right) + {\varTheta }(n\cdot \ell _2)\;\;\mathrm {and} \\ \text {E}\left( T_f\right)&= {\varOmega }\left( n \cdot e^{2(N-1)\beta d_1 (\ell _1-1)/\ell _1} \right) + {\varTheta }(n\cdot \ell _2) \end{aligned}$$

provided $\beta d_1/\ell _1,\; \beta d_2/\ell _2={\varOmega }(1)$ and *N* being a large enough constant.

The conditions $\beta d_1/\ell _1,\; \beta d_2/\ell _2={\varOmega }(1)$ are identical to those in Lemma 10: SSWM must have a selection strength $\beta$ strong enough such that the probability of accepting a move uphill (fitness difference of $d_1/\ell _1$ or $d_2/\ell _2$) is ${\varOmega }(1)$. This is a necessary and sensible condition as otherwise SSWM struggles to climb uphill.

The upper and lower bounds in Theorem 11 are not tight because of the terms $(\ell _1+1)/\ell _1$ and $(\ell _1-1)/\ell _1$ in the exponents, respectively. However, both these terms converge to 1 as $\ell _1$ grows. The running time, particularly the term $e^{2N\beta d_1 (\ell _1+1)/\ell _1}$, crucially depends on $\beta d_1$, the depth of the valley after scaling. Note that the condition $\beta d_1/\ell _1={\varOmega }(1)$ is equivalent to $\beta d_1 = {\varOmega }(\ell _1)$, hence Theorem 11 applies if the depth after scaling is at least of the same order of growth as the length (recall that $d_1$ and $\ell _1$ may grow with *n*).

Theorem 11 also indicates how to choose $\beta$ according to the valley function in hand, in order to meet the theorem’s condition and to minimise the (upper bounds on the) running time. One can always choose $\beta =\varepsilon \ell _1/d_1$ for some constant $\varepsilon >0$ and any valley structure (even when $\ell _1 = \omega (d_1)$). This way the theorem’s condition becomes $\beta d_1/\ell _1=\varepsilon$ and the running time simplifies to $O\left( n \cdot e^{2N\beta \varepsilon (\ell _1+1)} \right) + {\varTheta }(n\cdot \ell _2)$, where we can choose the constant $\varepsilon >0$ as small as we like. For $N=O(1)$ we can further simplify the runtime to $O\left( n \cdot e^{O(\ell _1)} \right) + {\varTheta }(n\cdot \ell _2)$. For all $\ell _1 \ge 2$ (and reasonable $\ell _2$) this is asymptotically smaller that the expected optimisation time of the ($1+1$) EA, which is at least ${\varOmega }(n^{\ell _1})={\varOmega }(e^{\ell _1 \ln n})$ (see Theorem 3).

#### Proof of Theorem 11

The first part of the proof consists of estimating $\text {E}\left( T_{1 \rightarrow \ell _1}\right)$ by using the statement of Lemma 7. Then we will check that the conditions from Lemma 5 are met and we will add the ${\varTheta }(\ell _2)$ term. Finally, we will take into account the time needed for a relevant step in the long path to obtain the *n* factor in the bounds (see Lemma 2).

As just described above we start considering $\text {E}\left( T_{1 \rightarrow \ell _1}\right)$ by using Lemma 7. Let us start with the upper bound.

$$\begin{aligned} \text {E}\left( T_{1 \rightarrow \ell _1}\right) =\;&O\left( \frac{1}{p^\mathrm {GR}_{1 \rightarrow \ell _1}} \cdot \left( \text {E}\left( T^\mathrm {GR}_{1 , \ell _1}\right) + \frac{1}{p_{0 \rightarrow 1}} \right) \right) . \end{aligned}$$

Using Lemma 10 we bound $p^\mathrm {GR}_{1 \rightarrow \ell _1}$ yielding

$$\begin{aligned} \text {E}\left( T_{1 \rightarrow \ell _1}\right) =\;&O\left( \frac{e^{2(N-1)\beta d_1}}{2(N-1)\beta d_1/\ell _1} \cdot \left( O(1) + \frac{1}{p_{0 \rightarrow 1}}\right) \right) . \end{aligned}$$

Since $p_\mathrm {fix}$ for ${\varDelta }f<0$ decreases when the parameters *N*, $\beta$ and $|{\varDelta }f|$ increase and $N\beta d_1/\ell _1={\varOmega }(1)$, we get $p^{-1}_{0 \rightarrow 1}={\varOmega }(1)$ and $O(1) + \frac{1}{p_{0 \rightarrow 1}} = O \left( \frac{1}{p_{0 \rightarrow 1}}\right)$. Hence,

$$\begin{aligned} \text {E}\left( T_{1 \rightarrow \ell _1}\right) =\;&O\left( \frac{e^{2(N-1)\beta d_1}}{2(N-1)\beta d_1/\ell _1} \cdot \frac{1}{p_{0 \rightarrow 1}}\right) . \end{aligned}$$

Using Lemma 9 to lower bound $p_{0 \rightarrow 1}$ we get

$$\begin{aligned} \text {E}\left( T_{1 \rightarrow \ell _1}\right) =\;&O\left( \frac{e^{2(N-1)\beta d_1}}{2(N-1)\beta d_1/\ell _1} \cdot \frac{e^{2N\beta d_1/\ell _1}}{2\beta \frac{d_1}{\ell _1}} \right) . \end{aligned}$$

Using $(N-1)\beta d_1/\ell _1={\varOmega }(1)$ and $\beta d_1/\ell _1={\varOmega }(1)$ both terms to the denominator are ${\varOmega }(1)$ leading to

$$\begin{aligned} \text {E}\left( T_{1 \rightarrow \ell _1}\right) = O\left( e^{2N\beta d_1(\ell _1+1)/\ell _1} \right) . \end{aligned}$$

We now consider the lower bound. Starting again from Lemmas 5 and 7 and bounding $p^\mathrm {GR}_{1 \rightarrow \ell _1}$ with Lemma 10

$$\begin{aligned} \text {E}\left( T_{1 \rightarrow \ell _1}\right) =\;&{\varOmega }\left( \frac{1}{p^\mathrm {GR}_{1 \rightarrow \ell _1}} \right) = {\varOmega }\left( \frac{e^{2(N-1)\beta d_1}-1}{e^{2(N-1)\beta d_1/\ell _1}} \right) \\ =\;&{\varOmega }\left( e^{2(N-1)\beta d_1 \frac{\ell _1-1}{\ell _1}} -\frac{1}{e^{2(N-1)\beta d_1/\ell _1}}\right) \\ =\;&{\varOmega }\left( e^{2(N-1)\beta d_1 \frac{\ell _1-1}{\ell _1}} \right) . \end{aligned}$$

Now we need to apply Lemma 5 to add the ${\varTheta }(\ell _2)$ term in both bounds. We start checking that all the conditions are satisfied. Firstly, since $p_\mathrm {fix}$ for ${\varDelta }f>0$ increases when the parameters (*N*, $\beta$ and ${\varDelta }f$) increase, then $N\beta d_2/\ell _2={\varOmega }(1)$ implies $p_{\ell _1 \rightarrow \ell _1+1}={\varOmega }(1)$. Analogously for $p_{\ell _1 \rightarrow \ell _1-1}$ with $N\beta d_1/\ell _1 = {\varOmega }(1)$ satisfying the first property. Secondly, property (ii) follows directly from Lemma 8 and the condition $N\beta d_2/\ell _2 = {\varOmega }(1)$. The third property is satisfied since for $N>1$ the acceptance probability is strictly increasing with ${\varDelta }f$. Considering the time for a relevant step from Lemma 2 completes the proof. $\square$

### Application to Metropolis

We now apply the framework from Sect. 3.2 to the Metropolis algorithm. Since the analysis follows very closely the one of SSWM the proofs for this subsection are provided in the appendix. We first cast Metropolis on Valley as a Gambler’s Ruin problem. Like SSWM, Metropolis can make use of its parameter $\alpha$ to accommodate the gradient of Valley.

#### Lemma 12

(Metropolis Gambler’s Ruin downhill) Consider a Gambler’s Ruin problem as described in Theorem 4 with starting dollars $n_1=1$ and $n_2=\ell -1$, and probabilities $p_1$ and $p_2$ dependant on Metropolis’s acceptance function as follows

$$\begin{aligned} p_1 = \frac{1}{2}\cdot e^{-\alpha {\varDelta }f} \;\;\;\;\;\;\;\;\;\;\;\ p_2 = \frac{1}{2} \end{aligned}$$

where ${\varDelta }f<0$ and $\alpha |{\varDelta }f|={\varOmega }(1)$. Then the winning probability of player one $P_1$ can be bounded as follows

$$\begin{aligned} \frac{-\alpha {\varDelta }f}{e^{-\alpha \ell {\varDelta }f}}&< P^{\mathrm {GR-Met}}_1 < \frac{e^{-\alpha {\varDelta }f}}{e^{-\alpha \ell {\varDelta }f}-1} \end{aligned}$$

and the expected duration of the game will be $\text {E}\left( T^\mathrm {GR}_{1 , \ell }\right) = O(1)$.

Lastly, we make use of the previous lemma and the framework presented in Sect. 3.2 to determine bounds on the runtime of Metropolis for Valley. Note that the required conditions are similar to those from Theorem 11 for the SSWM algorithm, with only difference being that the parameter $\alpha$ substitutes the selection strength $\beta$. Hence the previous considerations for SSWM translate to Metropolis on Valley by simply applying $\beta \leftarrow \alpha$.

#### Theorem 13

The expected number of function evaluations $\text {E}\left( T_f\right)$ for Metropolis to reach $P_{\ell _1+\ell _2}$ from $P_0$ on Valley with $\ell _1, \ell _2 \in {\mathbb N}{\setminus }\{1\}$ and $d_1, d_2 \in R^+$ is

$$\begin{aligned} E(T_f)&= O\left( n\cdot e^{ \alpha d_1(1+1/\ell _1)}\right) + {\varTheta }(n\cdot \ell _2)\;\;\mathrm {and}\\ E(T_f)&= {\varOmega }\left( n\cdot e^{\alpha d_1(1-1/\ell _1)} \right) + {\varTheta }(n\cdot \ell _2) \end{aligned}$$

provided $$\alpha d_1/\ell _1$$, $$\alpha d_2/\ell _2={\varOmega }(1)$$.

## Crossing Concatenated Valleys

**Fig. 3:**
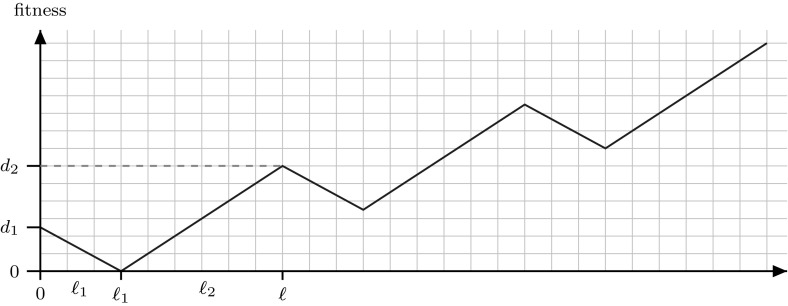
Sketch of the function ${\textsc {ValleyPath}}$

We define a class of functions called ValleyPath consisting of *m* consecutive valleys of the same size. Each of the consecutive valleys is shifted such that the fitness at the beginning of each valley is the same as that at the end of the previous valley (see Fig. 3). Fitness values from one valley to the next valley increase by an amount of ${d_2-d_1 > 0}$. Formally:

$$\begin{aligned} h(i,j)_{\textsc {ValleyPath}} := {\left\{ \begin{array}{ll} j \cdot (d_2 -d_1) + d_1 - i \cdot \frac{d_1}{\ell _1} &{} \text { if }i \le \ell _1\\ j \cdot (d_2 -d_1) + (i-\ell _1) \cdot \frac{d_2}{\ell _2} &{} \text { if }\ell _1 < i \le \ell . \end{array}\right. } \end{aligned}$$

Here $0< j \le m$ indicates a valley while $0\le i \le \ell _1+\ell _2=\ell$ indicates the position in the given valley. Hence, the global optimum is the path point ${{\mathrm{P}}}_{m\cdot \ell }$.

ValleyPath represents a rugged fitness landscape with many valleys and many local optima (peaks). It loosely resembles a “big valley” structure found in many real-world problems [1, 19, 22, 30]: from a high-level view the concatenation of valleys indicates a “global” gradient, i. e. the direction towards valleys at higher indices. The difficulty for optimisation algorithms is to overcome these many local optima and to still be able to identify the underlying gradient. We show here that both SSWM and Metropolis are able to exploit this global gradient and find the global optimum efficiently. Note that ValleyPath is a very broad function class in that it allows for many shapes to emerge, from few deep valleys to many shallow ones. Our results hold for all valley paths with $d_1<d_2$.

As in the analysis for Valley, instead of considering the whole Markov chain underlying ValleyPath we take a high-level view and consider the chain that describes transitions between neighbouring peaks. Since the peaks have increasing fitness, this chain is quite simple and allows for an easy application of drift arguments. By choosing the number of peaks to the right of the current peak as distance function, the next theorem shows that, if we can find constant bounds for the drift, we will only need to repeat the Valley experiment for as many peaks as there are in ValleyPath.

### Theorem 14

Consider any algorithm described by Algorithm 1 with local mutations on ValleyPath. Consider the points in time where the algorithm is on a peak and focus on transitions between different peaks. Let $X_t$ be a random variable describing the number of peaks to the right of the current valley at the *t*-th time a different peak is reached. If the drift over peaks ${\varDelta }$ can be lower bounded by some positive constant

9 $$\begin{aligned} {\varDelta }:= \text {E}\left( X_t - X_{t+1} \mid X_t>0\right) \ge c > 0 \end{aligned}$$

then the expected number of function evaluations $\text {E}\left( T_f\right)$ to reach the optimum starting from any peak is

$$\begin{aligned} \text {E}\left( T_f\right)&= O\left( m\cdot \text {E}\left( T^{O}_{\textsc {Valley}}\right) \right) \;\;\text {and}\;\; {\varOmega }\left( m\cdot \text {E}\left( T^{{\varOmega }}_{\textsc {Valley}}\right) \right) \end{aligned}$$

where *m* is the number of valleys that compose ValleyPath, and $\text {E}\left( T^{O}_{\textsc {Valley}}\right)$ and $\text {E}\left( T^{{\varOmega }}_{\textsc {Valley}}\right)$ are the upper and lower bounds for Valley respectively.

### Proof

The lower bound is trivial since the algorithm can only move to a neighbour peak and has to visit *m* peaks. The upper bound follows from the standard additive drift theorem [10, 18]. $\square$

To compute the drift over the peaks ${\varDelta }$ [see Eq. (9)] needed to use the previous theorem we perform a slightly different abstraction over the ValleyPath problem. We will also consider, apart from the peaks (local maxima), the points of minimal fitness between them (valleys). For simplicity we will use the following notation.

### Definition 3

( ValleyPath *Notation*) Consider any algorithm described by Algorithm 1 with local mutations where the current search point is any extreme point (a maximum or minimum) of ValleyPath. If the algorithm is on a valley (minimum) we will denote by:

1. $T_{\mathrm {peaks}}$ the first hitting time of either of the neighbouring peaks,
2. $p_r^\uparrow$ the probability of the algorithm being in the right-hand peak at $T_{\mathrm {peaks}}$,
3. $p_l^\uparrow =1-p_r^\uparrow$ the probability of the algorithm being in the left-hand peak at $T_{\mathrm {peaks}}$.

If the algorithm is on a peak (a maximum) we will denote by:

(4) $T_{\mathrm {min}}$ the first hitting time of either of the neighbouring minima,
(5) $p_r^\downarrow$ the probability of the algorithm being in the right-hand minimum at $T_{\mathrm {min}}$,
(6) $p_l^\downarrow =1-p_r^\downarrow$ the probability of the algorithm being in the left-hand minimum at $T_{\mathrm {min}}$.

**Fig. 4:**
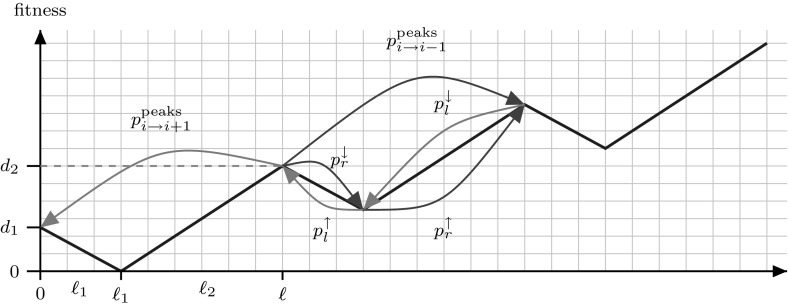
Sketch of the function ${\textsc {ValleyPath}}$ with the probabilities used for its runtime analysis

The following lemma computes the drift between peaks ${\varDelta }$ [Eq. (9)] by introducing transition probabilities between neighbouring peaks $p^{\mathrm {peaks}}_{i\rightarrow i-1}$ and $p^{\mathrm {peaks}}_{i\rightarrow i+1}$ (see Fig. 4). These two new probabilities can be expressed in terms of the transition probabilities between consecutive peaks and minima from Definition 3, yielding a neat expression for the drift.

### Lemma 15

In the context of Theorem 14 and Definition 3, if $p_r^\downarrow p_r^\uparrow \ge \gamma p_l^\downarrow p_l^\uparrow$ for some constant $\gamma >1$ then the drift between peaks will be ${\varOmega }(1)$.

### Proof

Let us start from the definition of the drift between peaks from Theorem 14, we expand the drift in terms of two probabilities: $p^{\mathrm {peaks}}_{i\rightarrow i-1}$ which reads as the probability of decreasing the number of peaks on the right by 1 and for the opposite event $p^{\mathrm {peaks}}_{i\rightarrow i+1}=1-p^{\mathrm {peaks}}_{i\rightarrow i-1}$. Then,

$$\begin{aligned} \text {E}\left( X_t - X_{t+1} \mid X_t=i >0\right)&= i - p^{\mathrm {peaks}}_{i\rightarrow i-1}(i-1) - p^{\mathrm {peaks}}_{i\rightarrow i+1} (i+1) \\&= i\cdot \left( 1-p^{\mathrm {peaks}}_{i\rightarrow i-1}-p^{\mathrm {peaks}}_{i\rightarrow i+1}\right) + p^{\mathrm {peaks}}_{i\rightarrow i-1} - p^{\mathrm {peaks}}_{i\rightarrow i+1} \\&= 2p^{\mathrm {peaks}}_{i\rightarrow i-1} - 1 \end{aligned}$$

where in the last step we have used $p^{\mathrm {peaks}}_{i\rightarrow i-1}+p^{\mathrm {peaks}}_{i\rightarrow i+1}=1$. Therefore a sufficient condition for the drift to be constant is $p^{\mathrm {peaks}}_{i\rightarrow i-1} \ge \frac{1}{2}+\frac{1}{c}$ for some constant $c\ge 2$.

We can break down this term using the four probabilities from Definition 3. We consider the Markov Chain composed of the extreme points (maxima and minima) of ValleyPath and the algorithm on a peak. After two steps the system can be only in one of three points: with probability $p_l^\downarrow p_l^\uparrow$ the algorithm will reach the peak on its left, analogously it will reach the right peak with probability $p_r^\downarrow p_r^\uparrow$ and with the remaining probability $p_r^\downarrow p_l^\uparrow + p_l^\downarrow p_r^\uparrow$ the algorithm will leave and return to the starting peak before reaching any other peak. We can now express the probability of moving to a specific peak given that we have moved to a peak $p^{\mathrm {peaks}}_{i\rightarrow i-1}$ as

$$\begin{aligned} p^{\mathrm {peaks}}_{i\rightarrow i-1}&= \frac{p_r^\downarrow p_r^\uparrow }{p_r^\downarrow p_r^\uparrow + p_l^\downarrow p_l^\uparrow } = \frac{1}{1+\frac{p_l^\downarrow p_l^\uparrow }{p_r^\downarrow p_r^\uparrow }}. \end{aligned}$$

The previous condition $p^{\mathrm {peaks}}_{i\rightarrow i-1} \ge \frac{1}{2}+\frac{1}{c}$ can now be translated to $p_r^\downarrow p_r^\uparrow \ge \gamma p_l^\downarrow p_l^\uparrow$ for some constant $\gamma >1$. $\square$

The previous lemma gives us a simple equation that determines the drift. Losing rigour for a moment we neglect the factor $\gamma$ and identify some regimes where the overall drift is positive: (1) $p_r^\downarrow > p_l^\downarrow$ and $p_r^\uparrow > p_l^\uparrow$, (2) $p_r^\downarrow \gg p_l^\downarrow$ and $p_r^\uparrow < p_l^\uparrow$ or (3) $p_r^\downarrow < p_l^\downarrow$ and $p_r^\uparrow \gg p_l^\uparrow$. In the next lemma we recover the original Markov Chain and express these four probabilities in terms of the real transition probabilities between the states of ValleyPath. We will also make an assumption on the algorithm (the probability of accepting an improvement of ${\varDelta }f$ must be exponentially bigger than the probability of accepting a worsening of the same size with ${\varDelta }f$). As the reader might notice from the previous section both SSWM and Metropolis meet this condition.

Finally we can simplify the condition to have a positive drift in a neat expression that only depends on the depth of the valleys ($d_1$ and $d_2$) and the parameter $\lambda$ of the acceptance probability distribution.

### Lemma 16

In the context of Lemma 15, consider any algorithm described by Algorithm 1 with an acceptance function such that $\frac{p_{\mathrm {acc}}({\varDelta }f)}{p_{\mathrm {acc}}(-{\varDelta }f)} = e^{\lambda {\varDelta }f}$ for some $\lambda \in {\mathbb R}^+$. Then

$$\begin{aligned} \frac{p_l^\downarrow p_l^\uparrow }{p_r^\downarrow p_r^\uparrow } = e^{-\lambda \left( d_2 - d_1 \right) }. \end{aligned}$$

### Proof

According to our notation (Definition 3) $p_l^\downarrow$ reads as the probability of reaching the minimum on the left of a peak before reaching the minimum on its right. As in the Valley section we can break down the process of moving to a neighbouring minimum in two steps: (1) first moving just one point towards the left slope and (2) winning a Gambler’s Ruin game starting with one dollar, using a notation in the same spirit as in the previous section (see Definition 2). We will respectively denote the probability of the events (1) and (2) $p_{\ell _2}^\downarrow$ and $p_{\ell _2}^{\mathrm {GR}\downarrow }$, where $\ell _2$ determines that the process is on the slope with length $\ell _2$. Using the same rationale for the other probabilities we can rewrite the quotient from Lemma 15 as

$$\begin{aligned} \frac{p_l^\downarrow p_l^\uparrow }{p_r^\downarrow p_r^\uparrow }&= \frac{ \left( \frac{p_{\ell _2}^\downarrow p_{\ell _2}^{\mathrm {GR}\downarrow }}{p_{\ell _2}^\downarrow p_{\ell _2}^{\mathrm {GR}\downarrow }+p_{\ell _1}^\downarrow p_{\ell _1}^{\mathrm {GR}\downarrow }}\right) \cdot \left( \frac{p_{\ell _1}^\uparrow p_{\ell _1}^{\mathrm {GR}\uparrow }}{p_{\ell _1}^\uparrow p_{\ell _1}^{\mathrm {GR}\uparrow }+p_{\ell _2}^\uparrow p_{\ell _2}^{\mathrm {GR}\uparrow }}\right) }{ \left( \frac{p_{\ell _1}^\downarrow p_{\ell _1}^{\mathrm {GR}\downarrow }}{p_{\ell _1}^\downarrow p_{\ell _1}^{\mathrm {GR}\downarrow }+p_{\ell _2}^\downarrow p_{\ell _2}^{\mathrm {GR}\downarrow }}\right) \cdot \left( \frac{p_{\ell _2}^\uparrow p_{\ell _2}^{\mathrm {GR}\uparrow }}{p_{\ell _1}^\uparrow p_{\ell _1}^{\mathrm {GR}\uparrow }+p_{\ell _2}^\uparrow p_{\ell _2}^{\mathrm {GR}\uparrow }}\right) }\\&= \frac{p_{\ell _2}^\downarrow p_{\ell _2}^{\mathrm {GR}\downarrow } \cdot p_{\ell _1}^\uparrow p_{\ell _1}^{\mathrm {GR}\uparrow }}{p_{\ell _1}^\downarrow p_{\ell _1}^{\mathrm {GR}\downarrow } \cdot p_{\ell _2}^\uparrow p_{\ell _2}^{\mathrm {GR}\uparrow }} \\&= \frac{p_{\ell _2}^\downarrow }{p_{\ell _2}^{\uparrow }}\cdot \frac{p_{\ell _1}^\uparrow }{p_{\ell _1}^{\downarrow }}\cdot \frac{p_{\ell _2}^{\mathrm {GR}\downarrow }}{p_{\ell _2}^{\mathrm {GR}\uparrow }}\cdot \frac{p_{\ell _1}^{\mathrm {GR}\uparrow }}{p_{\ell _1}^{\mathrm {GR}\downarrow }}. \end{aligned}$$

Using the Gambler’s Ruin problem results (see Theorem 4) we can expand the previous equality into

$$\begin{aligned} \frac{p_l^\downarrow p_l^\uparrow }{p_r^\downarrow p_r^\uparrow }&= \frac{p_{\ell _2}^\downarrow }{p_{\ell _2}^{\uparrow }}\cdot \frac{p_{\ell _1}^\uparrow }{p_{\ell _1}^{\downarrow }}\cdot \frac{\left( \frac{1-\frac{p_{\ell _2}^{\uparrow }}{p_{\ell _2}^{\downarrow }}}{1-\left( \frac{p_{\ell _2}^{\uparrow }}{p_{\ell _2}^{\downarrow }}\right) ^{\ell _2}}\right) }{\left( \frac{1-\frac{p_{\ell _2}^{\downarrow }}{p_{\ell _2}^{\uparrow }}}{1-\left( \frac{p_{\ell _2}^{\downarrow }}{p_{\ell _2}^{\uparrow }}\right) ^{\ell _2}}\right) }\cdot \frac{\left( \frac{1-\frac{p_{\ell _1}^{\downarrow }}{p_{\ell _1}^{\uparrow }}}{1-\left( \frac{p_{\ell _1}^{\downarrow }}{p_{\ell _1}^{\uparrow }}\right) ^{\ell _1}}\right) }{\left( \frac{1-\frac{p_{\ell _1}^{\uparrow }}{p_{\ell _1}^{\downarrow }}}{1-\left( \frac{p_{\ell _1}^{\uparrow }}{p_{\ell _1}^{\downarrow }}\right) ^{\ell _1}}\right) }. \end{aligned}$$

Now using the acceptance function’s property from the lemma statement we can simplify this expression to

$$\begin{aligned} \frac{p_l^\downarrow p_l^\uparrow }{p_r^\downarrow p_r^\uparrow }&= e^{-\lambda d_2/\ell _2}\cdot e^{\lambda d_1/\ell _1}\cdot \frac{\left( \frac{1-e^{\lambda d_2/\ell _2}}{1-e^{\lambda d_2}}\right) }{\left( \frac{1-e^{-\lambda d_2/\ell _2}}{1-e^{-\lambda d_2}}\right) } \cdot \frac{\left( \frac{1-e^{-\lambda d_1/\ell _1}}{1-e^{-\lambda d_1}}\right) }{\left( \frac{1-e^{\lambda d_1/\ell _1}}{1-e^{\lambda d_1}}\right) }. \end{aligned}$$

Notice that the last four terms have the same format as the equation for $p_\mathrm {fix}$ (with different parameters). Lets rename them as $p_\mathrm {fix}^{*}$ for simplicity

$$\begin{aligned} \frac{p_l^\downarrow p_l^\uparrow }{p_r^\downarrow p_r^\uparrow }&= e^{-\lambda d_2/\ell _2}\cdot e^{\lambda d_1/\ell _1}\cdot \left( \frac{p_\mathrm {fix}^{* \downarrow \ell _2}}{p_\mathrm {fix}^{* \uparrow \ell _2}} \right) \cdot \left( \frac{p_\mathrm {fix}^{* \uparrow \ell _1}}{p_\mathrm {fix}^{* \downarrow \ell _1}}\right) . \end{aligned}$$

We find again the ratio between symmetric fitness differences, then Lemma 8 highly simplifies the previous expression to

$$\begin{aligned} \frac{p_l^\downarrow p_l^\uparrow }{p_r^\downarrow p_r^\uparrow }&= e^{-\lambda d_2/\ell _2}\cdot e^{\lambda d_1/\ell _1}\cdot e^{-\lambda d_2 (\ell _2-1)/\ell _2} \cdot e^{\lambda d_1 (\ell _1-1)/\ell _1} \\&= e^{-\lambda \left( d_2 - d_1 \right) }. \end{aligned}$$

$$\square $$

### Application for SSWM and Metropolis

In the next two theorems we apply the previous results on ValleyPath to the SSWM and Metropolis algorithms. The application is straightforward when making the parameter $\lambda = {\varOmega }(1)$ and the depths of the valley ($d_1$ and $d_2$) differ in some positive constant. Notice that it could be the case that $d_2 - d_1$ is smaller than a constant but the parameters are big enough to compensate for this effect and still have a positive drift over peaks. However this increase in the parameters will affect the optimisation time between peaks (i.e. the Valley problem). Note that, by applying Theorem 3, it is easy to see that the runtime of the ($1+1$) EA will be exponential in the length of the individual valleys, hence the algorithm will be efficient only for valley paths consisting of valleys of moderate length.

The remaining conditions that Theorems 17 and 18 require are those already required on the analysis for Valley (see Theorems 11 and 13).

#### Theorem 17

The expected number of function evaluations $$\text {E}\left( T_f\right) $$ for SSWM to reach the optimum starting from any peak on ValleyPath with $$2\beta (N-1) \cdot ( d_2-d_1 ) \ge c $$ for some constant $$c >0 $$ is

$$\begin{aligned} \text {E}\left( T_f\right)&= O\left( m \cdot n \cdot \left( e^{2N\beta d_1 (l_1+1)/l_1} + {\varTheta }(l_2)\right) \right) \;\text {and}\\ \text {E}\left( T_f\right)&= {\varOmega }\left( m \cdot n \cdot \left( e^{2(N-1)\beta d_1 (l_1-1)/l_1} + {\varTheta }(l_2)\right) \right) \end{aligned}$$

provided $$\ell _1, \ell _2 \in {\mathbb N}{\setminus }\{1\}$$, $$d_2 > d_1 $$, $$N={\varOmega }(1)$$ and $$\beta d_1/\ell _1, \beta d_2 / \ell _2 = {\varOmega }(1)$$.

#### Proof

Due to Lemma 8, SSWM meets the exponential ratio property needed by Lemma 16 with $\lambda =2\beta (N-1)$. Then we can say that

$$\begin{aligned} \frac{p_l^\downarrow p_l^\uparrow }{p_r^\downarrow p_r^\uparrow } = e^{-\lambda (d_2-d_1)} = \frac{1}{e^{2\beta (N-1) ( d_2-d_1 )}} = \frac{1}{\gamma }. \end{aligned}$$

Since $2\beta (N-1) ( d_2-d_1 ) \ge c > 0$, then $\gamma$ is a constant greater than 1 fulfilling the condition required by Lemma 15 for the drift to be constant. Finally we apply Theorem 14 taking into account the optimisation time for Valley to obtain the claimed result. $\square$

An equivalent result to that of the SSWM for ValleyPath is shown for Metropolis in the following theorem.

#### Theorem 18

The expected number of function evaluations $$\text {E}\left( T_f\right) $$ for Metropolis to reach the optimum starting from any peak on ValleyPath with $$\alpha ( d_2-d_1 ) \ge c $$ for some constant $$c >0 $$ is

$$\begin{aligned} \text {E}\left( T_f\right) =\;&O\left( m \cdot n \cdot \left( e^{\alpha d_1 (l_1+1)/l_1}+ {\varTheta }(l_2)\right) \right) \;\text {and}\\ \text {E}\left( T_f\right) =\;&{\varOmega }\left( m \cdot n \cdot \left( e^{\alpha d_1 (l_1-1)/l_1}+ {\varTheta }(l_2)\right) \right) \end{aligned}$$

provided $$\ell _1, \ell _2 \in {\mathbb N}{\setminus }\{1\}$$, $$d_2 > d_1 $$, and $$\alpha d_1/\ell _1, \alpha d_2 / \ell _2 = {\varOmega }(1)$$.

#### Proof

The proof follows exactly as the proof of Theorem 17 with the only difference that $\lambda =\alpha$ [see Eq. (2)]. $\square$

Note that our approach can be extended to concatenations of valleys of different sizes, assuming $d_1<d_2$ for each valley. In this case the expression of the runtime would be dominated by the deepest valley.

## Conclusions

We presented an analysis of randomised search heuristics for crossing fitness valleys where no mutational bias exists and thus the probability for moving forwards or backwards on the path depends only on the fitness difference between neighbouring search points. Our focus was to highlight characteristics of valleys where an elitist selection strategy should be preferred to a non-elitist one and vice versa. In particular, we compared the ($1+1$) EA using standard bit mutation with elitism against two algorithms using local mutations with non-elitism, namely SSWM and Metropolis. To achieve our goals we presented a mathematical framework to allow the analysis of non-elitist algorithms on valleys and paths of concatenated valleys. We rigorously proved that while the ($1+1$) EA is efficient for valleys and valley paths up to moderate lengths, both SSWM and Metropolis are efficient when the valleys and valley paths are not too deep. A natural direction for future work is to extend the mathematical framework to allow the analysis of SSWM with global mutations, thus highlighting the benefits of combining both non-elitism and global mutations for overcoming local optima.

## A Omitted Proofs for Valley

This appendix contains proofs that were omitted from the main part.

### Restating Lemma 6

In the context of Lemma 5, properties (i) and (ii) imply that

(i) $p_{\ell _1 \rightarrow \ell _1-1}+p_{\ell _1 \rightarrow \ell _1+1} = 1/c_1$ for some constant $c_1\ge 1$
(ii) $1-c_1\cdot p_{\ell _1 \rightarrow \ell _1+1} \cdot p^\mathrm {GR}_{\ell _1+1 \rightarrow \ell _1} = 1/c_2$ for some constant $c_2>1$
(iii) $1-c_1c_2\cdot p_{\ell _1 \rightarrow \ell _1-1} = 1/c_3$ for some constant $c_3>1$

### Proof of Lemma 6

The first result follows directly from

$$\begin{aligned} p_{\ell _1 \rightarrow \ell _1+1},\; p_{\ell _1 \rightarrow \ell _1-1}&= {\varOmega }(1) \;\;\;\;&(\text {property (i) of Lemma }5) \\ p_{\ell _1 \rightarrow \ell _1-1}+p_{\ell _1 \rightarrow \ell _1+1}&\le 1 \end{aligned}$$

For the second result, first we observe that $1-c_1\cdot p_{\ell _1 \rightarrow \ell _1+1} \cdot p^\mathrm {GR}_{\ell _1+1 \rightarrow \ell _1} <1$. Then we need to prove a constant lower bound on $1-c_1\cdot p_{\ell _1 \rightarrow \ell _1+1} \cdot p^\mathrm {GR}_{\ell _1+1 \rightarrow \ell _1}$. For that, we start considering the term $p^\mathrm {GR}_{\ell _1+1 \rightarrow \ell _1}$, which was defined in the framework’s notation (see Definition 2). Using the results from the Gambler’s Ruin problem (see Theorem 4) we can express $p^\mathrm {GR}_{\ell _1+1 \rightarrow \ell _1}$ as

10 $$\begin{aligned} p^\mathrm {GR}_{\ell _1+1 \rightarrow \ell _1}= & {} \frac{1-\left( \frac{p_{\ell _1 \rightarrow \ell _1+1}}{p_{\ell _1+1 \rightarrow \ell _1}} \right) ^{\ell _2-1}}{ 1-\left( \frac{p_{\ell _1 \rightarrow \ell _1+1}}{p_{\ell _1+1 \rightarrow \ell _1}}\right) ^{\ell _2}} = \frac{p_{\ell _1+1 \rightarrow \ell _1}}{p_{\ell _1 \rightarrow \ell _1+1}}\cdot \frac{\left( \frac{p_{\ell _1 \rightarrow \ell _1+1}}{p_{\ell _1+1 \rightarrow \ell _1}} \right) ^{\ell _2}-\frac{p_{\ell _1 \rightarrow \ell _1+1}}{p_{\ell _1+1 \rightarrow \ell _1}}}{ \left( \frac{p_{\ell _1 \rightarrow \ell _1+1}}{p_{\ell _1+1 \rightarrow \ell _1}}\right) ^{\ell _2}-1} \nonumber \\\le & {} \frac{p_{\ell _1+1 \rightarrow \ell _1}}{p_{\ell _1 \rightarrow \ell _1+1}} \end{aligned}$$

where in the last step we have used that $p_{\ell _1+1 \rightarrow \ell _1} < p_{\ell _1 \rightarrow \ell _1+1}$ (property (ii) of Lemma 5) to upper bound the quotient by 1. Now we recover the second claim and introduce the already proven first result: $p_{\ell _1 \rightarrow \ell _1-1}+p_{\ell _1 \rightarrow \ell _1+1} = 1/c_1$ leading to

$$\begin{aligned} 1-c_1\cdot p_{\ell _1 \rightarrow \ell _1+1} \cdot p^\mathrm {GR}_{\ell _1+1 \rightarrow \ell _1}&= 1-\frac{p_{\ell _1 \rightarrow \ell _1+1} \cdot p^\mathrm {GR}_{\ell _1+1 \rightarrow \ell _1}}{p_{\ell _1 \rightarrow \ell _1-1}+p_{\ell _1 \rightarrow \ell _1+1}}. \end{aligned}$$

Using $p^\mathrm {GR}_{\ell _1+1 \rightarrow \ell _1}\le p_{\ell _1+1 \rightarrow \ell _1} / p_{\ell _1 \rightarrow \ell _1+1}$ obtained in Eq. (10) yields

$$\begin{aligned}1-c_1\cdot p_{\ell _1 \rightarrow \ell _1+1} \cdot p^\mathrm {GR}_{\ell _1+1 \rightarrow \ell _1}&\ge 1-\frac{ p_{\ell _1+1 \rightarrow \ell _1}}{p_{\ell _1 \rightarrow \ell _1+1}+p_{\ell _1 \rightarrow \ell _1-1}} \\&\ge \frac{p_{\ell _1 \rightarrow \ell _1+1}- p_{\ell _1+1 \rightarrow \ell _1}}{p_{\ell _1 \rightarrow \ell _1+1}+p_{\ell _1 \rightarrow \ell _1-1}} = \frac{1}{c} \end{aligned}$$

for some constant $c>1$. The last step follows since both numerator and denominator are constant due to properties (ii) and (i) from Lemma 5 respectively. The claimed equality from the statement with $1/c_2$ will hold for some constant $c_2$ in the range $c \ge c_2 > 1$.

Finally, for the third statement we start by observing that $1-c_1c_2\cdot p_{\ell _1 \rightarrow \ell _1-1} < 1$. The remaining part of the proof is to show a constant lower bound on $1-c_1c_2\cdot p_{\ell _1 \rightarrow \ell _1-1}$. For this, we introduce the already proven results for $c_1$ and $c_2$ obtaining

$$\begin{aligned}&1-c_1c_2\cdot p_{\ell _1 \rightarrow \ell _1-1} \\&\quad = 1-\frac{c_1\cdot p_{\ell _1 \rightarrow \ell _1-1}}{1-c_1\cdot p_{\ell _1 \rightarrow \ell _1+1} \cdot p^\mathrm {GR}_{\ell _1+1 \rightarrow \ell _1}} \\&\quad = 1-\frac{p_{\ell _1 \rightarrow \ell _1-1}}{p_{\ell _1 \rightarrow \ell _1-1}+p_{\ell _1 \rightarrow \ell _1+1}}\cdot \frac{1}{1-\frac{p_{\ell _1 \rightarrow \ell _1+1} \cdot p^\mathrm {GR}_{\ell _1+1 \rightarrow \ell _1}}{p_{\ell _1 \rightarrow \ell _1-1}+p_{\ell _1 \rightarrow \ell _1+1}}} \\&\quad = 1-\frac{p_{\ell _1 \rightarrow \ell _1-1}}{p_{\ell _1 \rightarrow \ell _1-1}+p_{\ell _1 \rightarrow \ell _1+1}-p_{\ell _1 \rightarrow \ell _1+1} \cdot p^\mathrm {GR}_{\ell _1+1 \rightarrow \ell _1}} \\&\quad \ge 1-\frac{p_{\ell _1 \rightarrow \ell _1-1}}{p_{\ell _1 \rightarrow \ell _1-1}+p_{\ell _1 \rightarrow \ell _1+1}-p_{\ell _1+1 \rightarrow \ell _1} }\;\;\;\; \qquad \qquad \qquad \qquad \qquad \qquad \qquad [\text {Eq.} (10)] \\&\quad \ge 1-\frac{p_{\ell _1 \rightarrow \ell _1-1}}{p_{\ell _1 \rightarrow \ell _1-1}+\varepsilon }, \;\; \varepsilon>0 \;\;\;\; \qquad \qquad \qquad \qquad \qquad \qquad (\text {property (ii) of Lemma} 5)\\&\quad = \frac{1}{c_3}, \;\; c_3>1. \; \qquad \qquad \qquad \qquad \qquad \qquad \qquad \qquad \qquad \qquad (\text {property (i) of Lemma} 5) \end{aligned}$$

$$\square $$

## B Analysis of Metropolis on Valley

### Restating Lemma 12

Consider a Gambler’s Ruin problem as described in Theorem 4 with starting dollars $n_1=1$ and $n_2=\ell -1$. And probabilities $p_1$ and $p_2$ dependant on Metropolis’s acceptance function as follows

$$\begin{aligned} p_1 = \frac{1}{2}\cdot e^{\alpha {\varDelta }f} \;\;\;\;\;\ p_2 = \frac{1}{2} \end{aligned}$$

where ${\varDelta }f<0$ and $\alpha |{\varDelta }f|={\varOmega }(1)$. Then the winning probability of player one $P_1$ can be bounded as follows

$$\begin{aligned} \frac{-\alpha {\varDelta }f}{e^{-\alpha \ell {\varDelta }f}}&< P^{\mathrm {GR-Met}}_1 < \frac{e^{-\alpha {\varDelta }f}}{e^{-\alpha \ell {\varDelta }f}-1} \end{aligned}$$

and the expected duration of the game will be $\text {E}\left( T^\mathrm {GR}_{1 , \ell }\right) = O(1)$.

### Proof of of Lemma 12

Since in general $1/2+ 1/2\cdot e^{-\alpha |{\varDelta }f|}< 1$ we have to make use of the Gambler’s Ruin problem with self-loops (see Theorem 4). Let us start with the winning probabilities

$$\begin{aligned} P^{\mathrm {GR-Met}}_{1\rightarrow \ell _1}&= \frac{1-\left( \frac{p_2}{p_1}\right) ^{n_1}}{1-\left( \frac{p_2}{p_1} \right) ^{n_1+n_2}} = \frac{1-e^{-\alpha n_1 {\varDelta }f}}{1-e^{-\alpha (n_1 +n_2) {\varDelta }f}}. \end{aligned}$$

For the upper bound:

$$\begin{aligned} P^{\mathrm {GR-Met}}_{1\rightarrow \ell _1}&= \frac{1-e^{-\alpha {\varDelta }f}}{1-e^{-\alpha \ell {\varDelta }f}} = \frac{e^{-\alpha {\varDelta }f}-1}{e^{-\alpha \ell {\varDelta }f}-1} <\;\frac{e^{-\alpha {\varDelta }f}}{e^{-\alpha \ell {\varDelta }f}-1}. \end{aligned}$$

For the lower bound:

$$\begin{aligned} P^{\mathrm {GR-Met}}_{1\rightarrow \ell _1}&= \frac{e^{-\alpha {\varDelta }f}-1}{e^{-\alpha \ell {\varDelta }f}-1}> \;\frac{-\alpha {\varDelta }f}{e^{-\alpha \ell {\varDelta }f}-1}> \frac{-\alpha {\varDelta }f}{e^{-\alpha \ell {\varDelta }f}}. \end{aligned}$$

Using again Theorem 4 for the expected duration of the game leads to

$$\begin{aligned} \text {E}\left( T^{\mathrm {GR-Met}}_{1,\ell _1}\right)&= \frac{ n_1 - (n_1+n_2)\cdot P^{\mathrm {GR-Met}}_1}{p_2^2-p_1^2} = \frac{1-\ell \cdot P^{\mathrm {GR-Met}}_1}{p_2^2-p_1^2} \le \frac{1}{p_2^2\left( 1-\frac{p_1^2}{p_2^2}\right) } \end{aligned}$$

introducing $p_1$ and $p_1$ and noticing that ${\varDelta }f < 0$ yields

$$\begin{aligned} \text {E}\left( T^{\mathrm {GR-Met}}_{1,\ell _1}\right)&\le \frac{4}{1-e^{2\alpha {\varDelta }f}} = \frac{4}{1-e^{-2\alpha |{\varDelta }f|}} = O(1). \end{aligned}$$

Where in the last step we have used that $\alpha |{\varDelta }f|={\varOmega }(1)$ implies $e^{-2\alpha |{\varDelta }f|}=1-{\varOmega }(1)$. $\square$

### Restating Theorem 13

The expected number of function evaluations $\text {E}\left( T_f\right)$ for Metropolis to reach $P_{\ell _1+\ell _2}$ from $P_0$ on Valley with $\ell _1, \ell _2 \in {\mathbb N}{\setminus }\{1\}$ and $d_1, d_2 \in R^+$ is

$$\begin{aligned} E(T)&= O\left( n\cdot e^{\alpha d_1(1+1/\ell _1)}\right) + {\varTheta }\left( n\cdot \ell _2 \right) \\ E(T)&= {\varOmega }\left( n\cdot e^{\alpha d_1(1-1/\ell _1)} \right) + {\varTheta }\left( n\cdot \ell _2 \right) \end{aligned}$$

provided $$\alpha d_1/\ell _1$$, $$\alpha d_2/\ell _2={\varOmega }(1)$$.

### Proof of of Theorem 13

Let’s begin by checking the conditions of Lemma 7 for Metropolis. First, *(i)* $p_{\ell _1\rightarrow \ell _1+1} =p_{\ell _1\rightarrow \ell _1-1} =1/2$. Next, *(ii)* $p_{\ell _1\rightarrow \ell _1+1}^2 = 1/4$ and $p_{\ell _1+1\rightarrow \ell _1} = e^{-\alpha d_2/\ell _2}/2$. This implies that condition (*ii*) only applies if $e^{-\alpha d_2/\ell _2}<\frac{1}{2}\Leftrightarrow \alpha d_2/\ell _2>\ln 2$. Conditions (*iii*) and (*iv*) are valid since Metropolis accepts solutions with a probability that depends only on ${\varDelta }f$ and is non-decreasing in ${\varDelta }f$. The proof for the fourth condition follows directly from $p_{\ell _1\rightarrow \ell _1+1} / p_{\ell _1+1\rightarrow \ell _1} = e^{\alpha d_2/\ell _2}$ and the condition $\alpha d_2/\ell _2 = {\varOmega }(1)$. Since these conditions are satisfied, Lemmas 5 and 6 apply and the expected time is:

$$\begin{aligned} \text {E}\left( T_{0 \rightarrow \ell _1+\ell _2}\right)&= {\varTheta }\left( \frac{1}{p^\mathrm {GR}_{1 \rightarrow \ell _1}} \cdot \left( \text {E}\left( T^\mathrm {GR}_{1 , \ell _1}\right) + \frac{p^\mathrm {GR}_{1 \rightarrow 0}}{p_{0 \rightarrow 1}} \right) \right) . \end{aligned}$$

For the upper bound, note that from Lemma 12:

$$\begin{aligned} \text {E}\left( T^{\mathrm {GR-Met}}_{1,\ell _1}\right)&=O\left( 1\right) \end{aligned}$$

as long as $\frac{\alpha d_1}{\ell _1}={\varOmega }(1)$. Then, using the bounds on $P_1$ from Lemma 12

$$\begin{aligned} \text {E}\left( T_{0 \rightarrow \ell _1+\ell _2}\right)&= O\left( \frac{1}{p^\mathrm {GR}_{1 \rightarrow \ell _1}} \cdot \left( \text {E}\left( T^\mathrm {GR-Met}_{1 , \ell _1}\right) + \frac{p^\mathrm {GR}_{1 \rightarrow 0}}{p_{0 \rightarrow 1}} \right) \right) \\&= O\left( \frac{1}{p^\mathrm {GR}_{1 \rightarrow \ell _1}} \cdot \left( O(1) + e^{\alpha d_1/\ell _1} \right) \right) \\&= O\left( \frac{ \ell _1 }{\alpha d_1} e^{\alpha d_1}\cdot \left( O(1) + e^{\alpha d_1/\ell _1} \right) \right) \\&= O\left( \frac{ \ell _1 }{\alpha d_1} e^{\alpha d_1(1+1/\ell _1)} \right) \\&= O\left( e^{\alpha d_1(1+1/\ell _1)} \right) . \end{aligned}$$

For the lower bound, since $\text {E}\left( T^\mathrm {GR-Met}_{1 , \ell _1}\right) >0$ and $\frac{p^\mathrm {GR}_{1 \rightarrow 0}}{p_{0 \rightarrow 1}}=e^{\alpha d_1/\ell _1}>1$ :

$$\begin{aligned} \text {E}\left( T_{0 \rightarrow \ell _1+\ell _2}\right)&= {\varOmega }\left( \frac{1}{p^\mathrm {GR}_{1 \rightarrow \ell _1}} \cdot \left( \text {E}\left( T^\mathrm {GR-Met}_{1 , \ell _1}\right) + \frac{p^\mathrm {GR}_{1 \rightarrow 0}}{p_{0 \rightarrow 1}} \right) \right) \\&= {\varOmega }\left( \frac{1}{p^\mathrm {GR}_{1 \rightarrow \ell _1}}\right) = {\varOmega }\left( \frac{e^{\alpha d_1}-1}{e^{\alpha d_1/\ell _1}} \right) \\&= {\varOmega }\left( e^{\alpha d_1(1-1/\ell _1)}-e^{-\alpha d_1/\ell _1}\right) \\&= {\varOmega }\left( e^{\alpha d_1(1-1/\ell _1)} \right) . \end{aligned}$$

Finally, we add the ${\varTheta }\left( \ell _2 \right)$ term (Lemma 5) and multiply by the time needed for a relevant step *n* / 2 (Lemma 2). $\square$
